# Health Promoting Properties of Cereal Vinegars

**DOI:** 10.3390/foods10020344

**Published:** 2021-02-05

**Authors:** Panagiotis Kandylis, Argyro Bekatorou, Dimitra Dimitrellou, Iris Plioni, Kanella Giannopoulou

**Affiliations:** 1 Department of Food Science and Technology, School of Agriculture, Aristotle University of Thessaloniki, P.O. Box 235, 54124 Thessaloniki, Greece; pkandylis@agro.auth.gr; 2 Department of Chemistry, University of Patras, 26504 Patras, Greece; dimitrellou@gmail.com (D.D.); plioni@upatras.gr (I.P.); kanellagiannop95@gmail.com (K.G.); 3 Department of Food Science and Technology, Ionian University, 28100 Argostoli, Kefalonia, Greece

**Keywords:** cereal vinegars, health properties, bioactive components, antioxidant, antimicrobial, antidiabetic, antiobesity, anticancer, antihypertensive

## Abstract

Vinegar has been used for its health promoting properties since antiquity. Nowadays, these properties are investigated, scientifically documented, and highlighted. The health benefits of vinegar have been associated with the presence of a variety of bioactive components such as acetic acid and other organic acids, phenolic compounds, amino acids, carotenoids, phytosterols, vitamins, minerals, and alkaloids, etc. These components are known to induce responses in the human body, such as antioxidant, antidiabetic, antimicrobial, antitumor, antiobesity, antihypertensive, and anti-inflammatory effects. The diversity and levels of bioactive components in vinegars depend on the raw material and the production method used. Cereal vinegars, which are more common in the Asia-Pacific region, are usually made from rice, although other cereals, such as millet, sorghum, barley, malt, wheat, corn, rye, oats, bran and chaff, are also used. A variety of bioactive components, such as organic acids, polyphenols, amino acids, vitamins, minerals, alkaloids, melanoidins, butenolides, and specific compounds such as γ-oryzanol, tetramethylpyrazine, γ-aminobutyric acid, etc., have been associated with the health properties of cereal vinegars. In this work, the bioactive components and the related health effects of cereal vinegars are reviewed, and the most recent scientific literature is presented and discussed.

## 1. Introduction

Vinegar is a liquid fermented product with at least 4% acetic acid content. It is consumed worldwide, either directly or as a food condiment [1,2]. The vinegar production process is often referred to as “vinegar brewing”. There are a vast variety of raw materials that can be used for vinegar production, including fruit (e.g., apples, grapes, dates, figs, plums, cherries, persimmon, etc.), cereals (e.g., rice, sorghum, barley, malt, wheat, corn, rye, oats), sugarcane, honey, coconut, roots and tubers (e.g., sweet potatoes), whey, and any other material that contains fermentable or hydrolysable carbohydrates [1,2,3].

Cereal vinegars can be produced through submerged or solid-state fermentation. The fermentation of the raw material generally involves three main stages: saccharification of starch, alcoholic fermentation of fermentable sugars, and oxidative fermentation (acetification) of ethanol into acetic acid [4]. The fermentation can be followed by an aging period. The core microbiota of the cereal vinegar fermentation is mainly represented by *Aspergillus* spp., lactic acid bacteria, acetic acid bacteria, and various *Saccharomyces* and non-*Saccharomyces* yeasts [5,6,7]. These microorganisms contribute to the enzymatic breakdown of the raw material and the production of bioactive molecules.

Vinegar has been used for its health promoting properties for centuries. These properties are nowadays investigated, scientifically documented, and highlighted. The health benefits of vinegar have been associated with the presence of a variety of bioactive components, such as acetic acid, phenolic acids, flavonoids, anthocyanins, amino acids, carotenoids, alkaloids, phytosterols, and vitamins, etc. (Table 1). These compounds are known to induce responses in the human body, such as antioxidative, antidiabetic, antimicrobial, antitumor, antiobesity, antihypertensive, anti-inflammatory, anti-aging, and cholesterol-regulating effects. Additional effects of vinegar consumption include alleviation of digestion, appetite, and fatigue conditions, etc. [8,9]. The diversity and content of bioactive compounds in vinegars depend on both the raw material and the production method used. The majority of cereal vinegars worldwide are produced from rice, as well as rice bran. Rice bran is rich in bioactive compounds (e.g., phenolic acids, γ-oryzanol, etc.) [10,11,12,13], while glutinous rice contains higher amounts of bioactive components compared to white rice [14].

Cereal vinegars are very common in Asian countries. Most Asian vinegars are made from rice, although other cereals such as millet, sorghum, barley, and chaff are also used. Chinese rice vinegars (RVs) vary in color from clear to black. The highest quality black Chinese vinegars are usually made from black glutinous (sticky) rice through a stationary surface fermentation (e.g., those produced in the Zhenjiang area), while in other areas (e.g., in Shanxi), cereals such as millet, sorghum, peas, barley, bran, and chaff are also used. The taste of black vinegars is milder and less sour than common RVs. The red Chinese RVs are stronger and owe their pigmentation and special organoleptic characteristics to the red mold *M. purpureus* that is involved in their manufacturing [2,15].

Generally, RVs can be made from white or brown, glutinous or non-glutinous rice, a saccharification fungal starter, and water. Unlike traditional black RV production, which is carried out by saccharification, alcoholic fermentation, and acetification in one step (in pots), RVs are nowadays usually made using Koji molds (*Aspergillus* spp. grown on steamed rice) with separate saccharification and fermentation steps (in stainless-steel or wooden tanks). The species involved in vinegar fermentations include fungi (mainly *Aspergillus* and *Rhizopus* spp.), yeasts (*Saccharomyces* and others), lactic acid bacteria, and acetic acid bacteria [2].

Malt vinegar (MV), on the other hand, is made by the fermentation of malted barley (germinated under controlled conditions) and is more popular in beer or whiskey producing countries (Europe, USA, Canada, and Australia). MV production involves alcoholic fermentation and acetification of malted barley, with or without the addition of other modified (partially saccharified) cereals [2]. MVs are bitter, and contain higher amounts of lactic acid compared to cider vinegars, and do not contain tartaric or malic acid.

Malted rice is also gaining increased attention for vinegar production in Asia. It is a highly nutritious raw material, with a variety of bioactive components, such as polyphenols, dietary fiber, tocopherols, γ-oryzanol, vitamins (thiamine, pyridoxine) and γ-aminobutyric acid (GABA), and with low levels of antinutrients such as phytic acid [2].

In this work, the bioactive components and the related health effects of cereal vinegars are reviewed, and the most recent scientific literature is presented and discussed.

## 2. Health Benefits of Cereal Vinegars

### 2.1. Antioxidant Properties

#### 2.1.1. Antioxidant Components

It is well established that several inflammatory, chronic, and degenerative diseases, including accelerated aging, are linked to oxidative stress, which can be described as the imbalance between pro-oxidant and antioxidant species in the human body [49]. Reactive species, such as reactive oxygen species (ROS; hydroxyl and superoxide radicals) and reactive nitrogen species (RNS; nitric oxide, peroxynitrite radicals), are necessary in limited amounts to ensure cell homeostasis, redox signaling and several essential cell functions such as gene expression, pathogen recognition, etc. However, excessive amounts, combined with insufficient antioxidant defense, will disrupt the redox equilibrium causing undesirable oxidative alterations in key biomolecules (DNA, proteins, lipids) and cell functions [49]. Apart from endogenous biochemical mechanisms, oxidative stress in the human body may be caused by exogenous factors such as smoking, environmental pollution and radiation, as well as by low intake of food antioxidants. Diets rich in antioxidant compounds can reduce degenerative health effects caused by reactive species, such as brain disorders, accelerated aging, and cancer [8,9,49,50].

**Figure 1:**
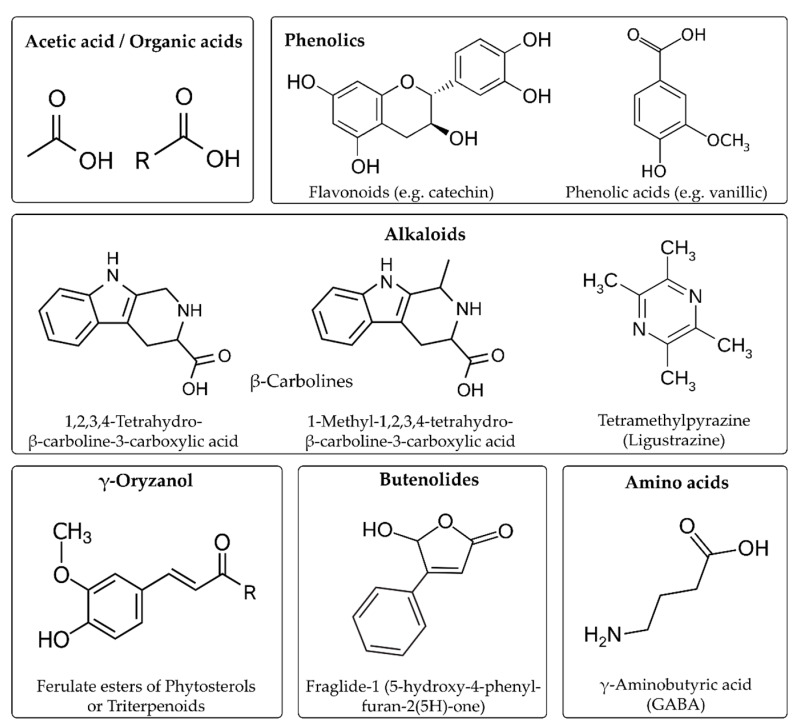
Important bioactive components in cereals and cereal vinegars.

Antioxidant bioactive components found in vinegars that can reduce such degenerative effects, include carotenoids, phytosterols, polyphenols (anthocyanins, flavonoids, phenolic acids), antioxidant vitamins (C and E), and other components, such as alkaloids, phytic acid, butenolides, GABA, ligustrazine (2,3,5,6-tetramethylpyrazine), melanoidins, etc., as shown in Table 1 and Figure 1.

Among bioactive compounds found in cereals and cereal vinegars, melanoidins alkaloids and butenolides (Figure 1) have recently attracted attention. In a study by Chiu et al. [27], the antioxidant, anti-mutagenic, and hypouricemic activities of sorghum vinegar (SoV) and Mei (plum) vinegar were evaluated and compared. Acetic acid, minerals, and alkaloid (β-carbolines) contents were found to be much higher in the Mei vinegar compared to SoV. Mei vinegar also presented higher xanthine oxidase inhibition activity (IC50 0.67 μg/mL in Mei vinegar and 0.96 μg/mL in SoV) that supports its potential antihyperuricemic effect. Moreover, the antioxidant capacity (AC) and solubility of urate were substantially ameliorated in Mei vinegar. The study further confirmed the hypothesis that has been pointed out by several other studies that alkaloids such as β-carbolines, which are found in both fruit and grain vinegars, largely contribute to antioxidant, antimutagenic, and hypouricemic effects [27].

Another antioxidant compound found in sticky rice husks and generated during vinegar production is 5-hydroxy-4-phenyl-butenolide (Fraglide-1). Butenolide compounds are known to have several bioactive properties (Table 1). Fraglide-1 was found in the Chinese ZAV Kozu and was shown to be an agonist for peroxisome proliferator-activated receptor γ (PPARγ; associated with the regulation of fatty acid storage and glucose metabolism) at the cellular level [47].

Melanoidins are another class of compounds that are considered to have a major contribution to the AC of vinegars. Liu et al. [51] suggested that melanoidin non-covalently bound compounds play a determinant role in the AC of vinegar melanoidins and could serve as targets for the optimization of the AC of vinegar melanoidins and vinegar as a whole. Specifically, it was found that the melanoidin non-covalently bound compounds from ZAV contributed 89% of the cellular AC of vinegar melanoidins and provided at least 85% of the bioavailable vinegar melanoidin phenolics to the proximal gastrointestinal tract [51].

Finally, γ-oryzanol, a rice bran constituent, which consists of a mixture of triterpene alcohol and phytosterol ferulates, also presents several health-promoting properties (anticarginogenic, anti-inflammatory, antihyperlipidemic, and neuroprotective), which have mainly been attributed to its significant AC [10,11,12,13].

While most of the earlier scientific studies describe the determination of antioxidant compounds in vinegars and their AC in vitro, most of the recent literature investigated in vivo the health effects of vinegar consumption [8].

#### 2.1.2. Antioxidant Activity and Protection against Oxidative Stress In Vitro and In Vivo

Regarding RVs, those made from unpolished rice and rice bran exhibit much higher AC compared to common rice vinegars. For example, the high radical scavenging capacity of the traditional Japanese vinegar Kurosu (or Kurozu; KuV)) was attributed to the significant amounts of phenolic acids (dihydroferulic and dihydrosinapic), which were extracted from the bran during fermentation [52,53,54]. The antioxidative activities of dihydroferulic acid (IC50: 15.1 μg/mL) and dihydrosinapic acid (IC50: 10.0 μg/mL) were stronger than those of ferulic acid (IC50: 22.1 µg/mL) and sinapic acid (IC50: 17.3 µg/mL), respectively [52]. KuV, as well as its ethyl acetate extracts, were found to be capable of inhibiting the growth of human cancer cells (breast, lung, colon, bladder, and prostate) in vitro (mainly in Caco-2 cells; up to 62% inhibition at a 0.025% KuV extract dose level) [55]. The KuV extracts also increased the glutathione S-transferase and quinone reductase activities and suppressed the formation of aberrant crypt foci (precursor lesion for colon adenocarcinoma) in rats [53]. The AC of a new type of vinegar, Izumi, made from unpolished rice by an improvement of the traditional method of KuV and containing higher levels of amino acids, was also evaluated by the determination of diacron-reactive oxygen metabolites (d-ROMs), biological antioxidant potential (BAP), red blood cell (RBC) deformability, and blood filtration time (BFT) [56]. Healthy, untrained female subjects consumed amounts of Izumi daily for up to 2 months. The results show no significant changes in weight, body mass index (BMI), fat mass, fat-free mass, peripheral blood variables, daily energy consumption, physical activity, and nutritional intake. On the other hand, the serum BAP level was increased significantly after 30 days of Izumi consumption, while the serum d-ROM levels and BFT were decreased. These results suggest that Izumi has increased AC compared to the traditional KuV and can reduce the oxidative stress and BFT in female subjects [56].

The organic acids, flavonoids, total phenolics, and AC of Chinese cereal vinegars were found to increase during their aging process [18,57]. Shanxi aged vinegar (SAV), a famous Chinese type of vinegar produced by solid-state fermentation, is known for its antioxidant properties as shown in several recent studies. It is also rich in bioactive compounds, such as proteins, fats, carbohydrates, organic acids, and amino acids that evolve during aging [24,57]. The correlation between total AC and total phenolic (TPC) and flavonoid contents (TFC) of SAV was investigated during its brewing process, and it was found that all parameters increased with aging time [24]. Specifically, the correlation coefficients between AC and TPC, when analyzed by the 2,2′-azino-bis-(3-ethylbenozthiazoline-6-sulphonic acid) radical scavenging (ABTS) and the ferric reducing antioxidant power (FRAP) assays, were 0.87 and 0.93, respectively. The correlation coefficients between AC and TFC were 0.83 (ABTS) and 0.88 (FRAP). At the stage of “smoking Pei” (i.e., the mash of cereal and husk after the alcoholic fermentation, which is used for the subsequent vinegar fermentation), the AC increased by 120% (ABTS) and 111% (FRAP) due to the increase in the TPC (89%) and TFC contents. Analysis of individual phenolics during the brewing process, showed that besides catechins and chlorogenic acid, gallic acid was a major antioxidant component. However, a minor loss of AC was observed after the 7th year. In addition, it was shown that the smoking Pei technique is important for enhancing the AC of the vinegar, therefore it should be further studied and controlled [24]. Likewise, the TPC, TFC, browning index, and AC of SAV increased with aging, as reported by Xia et al. [57], while the contribution of polyphenols and high molecular weight melanoidins to the total AC was similar (49% and 48%, respectively). The increase in phenolic compounds during aging was attributed to the concentration of vinegar by evaporation or ice removal, as well as to the hydrolysis of complex compounds (e.g., tannins), which generates small phenolic molecules (e.g., gallic acid). The phenolic compound dynamics may also be affected by their co-precipitation with other macromolecules during the brewing and aging processes, as in the case of rutin reduction that was observed in SAV [24].

In another study [58], the physicochemical properties of Fujian Yongchun aged vinegar (produced by submerged fermentation) and SAV (produced by solid-state fermentation) were compared with regard to the fermentation techniques and the aging periods applied (3–10 years). SAV presented higher pH, Brix, soluble solids, TPC, and AC values, but had lower total acidity and total organic acids content. It was also observed that the aging time increased pH, TPC, and AC. Based on the physicochemical characteristics and AC results, it was concluded that solid-state fermentation and the aging process are good brewing practices for improved vinegar quality [58].

SAV was also evaluated regarding its protective effects on ethanol-induced liver injury, and the results show that it could attenuate the hepatotoxicity in human L02 cells (immortalized hepatic cell line) and in mouse livers [39]. Specifically, an amount of 2.5 g vinegar/kg body weight was found to significantly decrease the ethanol-induced ROS and inhibit cell apoptosis and malonaldehyde levels in mouse livers. The improvement of the ethanol-induced oxidative stress and inflammation was also attributed to the downregulation of the expression of cytochrome P450 2E1 enzyme and NADPH oxidase (NOX). Therefore, SAV was characterized as a health-promoting hepatoprotective, antioxidant product, due to its ability to alleviate ethanol-induced oxidative stress [39]. Likewise, it was found to be able to improve the symptoms of oxidative stress and inflammation, induced by long-term high-fat diets, and improved gut microbiota disorders through decreasing the *Firmicutes*/*Bacteroidetes* ratio and increasing the relative abundance of beneficial bacteria [38]. These effects were attributed to the vinegar polyphenols (18 phenolic acids and 17 polyphenols were identified). The study concluded that consumption of SAV may be a novel strategy to alleviate the symptoms of oxidative stress and inflammation, as well as to restore the normal gut microbiota ecology in the treatment of metabolic syndromes [38].

Zhenjiang aromatic vinegar (ZAV) is another famous fermented Chinese product, produced by solid-state fermentation, and classified into traditional and industrial vinegar [18]. The variations in the chemical composition (proximate composition, organic acids, TPC, TFC, individual phenolics) and AC of these two types of ZAV were evaluated during their aging process. It was found that the contents of organic acids (mainly acetic, lactic and pyroglutamic), TPC, TFC, and AC were increased during aging. The proximate composition and organic acid contents were similar in both types of vinegars. However, the TPC, TFC and AC were higher in the traditional ZAV after 3 years of aging, while rutin and p-coumaric acid were detected only in traditional ZAV [18].

The changes in physicochemical properties, TPC, TFC, and AC of the traditional ZAV during its brewing process were also evaluated by Duan et al. [31], and the correlation between the TPC and AC was investigated. The results show that total acids, non-volatile acids, and amino nitrogen increased during the brewing process, while TPC, TFC, and AC were kept at low levels during alcoholic fermentation and reached their highest levels at the 6th year of aging. AC was correlated with both the TPC and TFC levels. p-hydroxybenzoic acid, vanillic acid, and catechin were the major phenolic compounds and also reached their highest levels after 6 years. Gallic, ferulic, and sinapic acids contributed more at the early stages of the aging process and then their contents declined. Catechin, vanillic acid, and syringic acid contributed more during the aging process. According to these findings, it was concluded that the quality and functional properties of ZAV could be controlled [31].

Xiangxi flavor vinegar (XFV) is another traditional Chinese vinegar (Hunan Province) made from herbs, rice, and spring water by spontaneous submerged fermentation. Its AC was investigated by analysis of antioxidant compounds, free radical scavenging capacity in vitro and in vivo, and the effects on antioxidant enzyme activity and apoptosis in *Caenorhabditis elegans* [35]. The results show that XFV is rich in antioxidant compounds such as ligustrazine (6.4 μg/mL). The in vitro 2,2-diphenyl-1- picrylhydrazyl free radical (DPPH•), hydroxyl radical (•OH), and superoxide anion radical (O_2_^−^) scavenging rates were 95.9%, 97.2%, and 63.3%, respectively, while the ROS content in *C. elegans* treated with the XFV was decreased significantly. Glutathione peroxidase, superoxide dismutase, and catalase activities were also significantly increased in *C. elegans*, while XFV upregulated the expression of the apoptosis regulator CED-9 protein and downregulated the CED-3 protein (a programmed cell death pathway protein). These results indicate that XFV is rich in antioxidant components able to effectively scavenge free radicals in vitro, while it can inhibit cell apoptosis, probably through the scavenging of ROS and the increase in antioxidant enzyme activities [35].

The AC of fermented Korean dark vinegar, made from unpolished rice, Nuruk starter, steamed rice cakes (Baekseolgi) and water, was also assessed [59]. The AC after 1 and 3 years was evaluated by the free radical scavenging method, and was found to be similar to that of 1 mg/mL ascorbic acid [59]. Several studies have also investigated the effects of black vinegar against metabolic illnesses in vivo. For example, black vinegar powder (BVP) is abundant in essential and hydrophobic amino acids (Table 1), catechin, chlorogenic acid, and minerals (K, Mg, Mn, Se), and has a considerable AC, as shown by Liu et al. [15]. Supplementation with BVP was found to increase the hepatic AC and lower the serum/liver thiobarbituric acid reactive substance values (TBARS lipid oxidation AC assay) in high-fat diet-fed rats [15].

The dynamic changes in AC and phenolic acid profiles of oat (OaV) and buckwheat vinegars (BuV) during different production stages were also evaluated and compared in vitro and in vivo [60]. The results show that both vinegars similarly attenuated the oxidative damage induced by D-galactose in mice serum and liver, in a non-dose dependent manner. However, OaV presented better in vitro AC than BuV. Moreover, the processes of alcoholic fermentation, acetous fermentation and fumigation induced a successive increase in the AC and phenolic acid content. Furthermore, the different fermentation processes applied led to a dynamic migration and transformation of specific phenolic acids across bound, esterified, and free fractions in the two types of vinegar. These findings indicate that the AC could be improved by targeted regulation of the generation of these fractions during the production processes [60].

Several methods for cereal vinegar production by novel raw materials and brewing techniques have been proposed, and the products were evaluated for their AC among other characteristics. For example, Pazuch et al. [61] proposed a method for defatted rice bran vinegar (BrV) production by submerged fermentation with optimized aeration and stirring conditions. Based on the AC and organoleptic properties of the products, it was concluded that defatted rice bran can be used as raw material for vinegar production. BrV contained 2.3 g/L of the natural plant antioxidant phytic acid, which corresponds to one third of that found in defatted rice bran and extracted during fermentation. BrV had an AC of 10.6 ± 1.0 μg/mL EC50 (median effective concentration in DPPH scavenging analysis), which is a significant result given that KuV (made from unpolished rice and rice bran) and polished rice vinegar have ACs of about 1710 and 3340 μg/mL EC50, respectively [61]. Broken Riceberry rice was also successfully used as a substrate for vinegar production by amylase saccharification, alcoholic fermentation by *Saccharomyces cerevisiae* and acetification by *Acetobacter aceti.* The vinegar contained about 11 mg/L anthocyanins and had an AC of about 966 mL of sample/g DPPH radical [37]. Likewise, the GABA and total anthocyanin content and the AC of vinegar brewed from germinated pigmented rice was studied by Phuapaiboon [29]. Four types of germinated rice were used: Hom Nil (purple rice), Riceberry (purple rice), Hom Mali Daeng (red rice) and control rice (ungerminated Riceberry rice), which were saccharified by red yeast rice, α-amylase and glucoamylase, and fermented by *S. cerevisiae* and *Acetobacter pasteurianum.* The concentration of GABA in the samples was in the range 1.14–1.73 mg/L; the highest being that of the germinated Hom Nil rice. The germination process decreased the total anthocyanin content, and all vinegars presented an AC, especially those from germinated Riceberry rice and germinated Hom Mali Daeng rice [29]. In a similar manner, uncooked germinated Tartary buckwheat (*Fagopyrum tataricum*; a type of pseudocereal similar to common buckwheat) was proposed for vinegar production (TBV) using the traditional technology of SAV [62]. TBV contained considerable amounts of TPC, TFC, GABA, and D-chiro-inositol, and had a considerable AC. It could also prolong the prothrombin time value and increase the radius of fibrinolytic ring significantly, which play an important thrombolytic and antithrombotic role [62]. A rich in polyphenols, alcohol-free type of brown vinegar made from beer (100% malted barley and two types of hops) was also proposed [63]. Vinegar brewing was carried out using the Orleans method (surface fermentation) with naturally occurring *A. aceti*. Both beer and vinegar samples were analyzed for phenolic content and AC as well as for individual phenolic compounds. The TPC of the beer and vinegar samples were about 429 and 662 mg GAE/L, respectively, and the AC (radical scavenging) was 63.0% and 82.2%, respectively. These values were comparable with those reported for beer and balsamic vinegars in other studies, while the acetic acid fermentation improved both the TPC and AC values. However, these parameters were not attributed solely to phenolic compounds, since the Folin–Ciocalteu reaction for TPC determination and the AC assay may be affected by beer and hops components, such as Maillard reaction products, sulphites, and xanthohumol. Based on the results, the new brown beer vinegar was proposed as an enriched source of valuable bioactive compounds compared to beer, which could also be of special diet interest [63]. Finally, the use of Leum Pua glutinous rice as substrate for vinegar production, after hydrolysis by a raw starch degrading enzyme (from *Laceyella sacchari* LP175), was evaluated by Lomthong and Saithong [14]. A vinegar with 5.7% acetic acid content and AC of 1218.8 ± 11.2 mL sample/g DPPH was obtained. However, the AC was decreased by about 30% as compared to the wine at the beginning of the acetous fermentation) [14].

### 2.2. Antimicrobial Properties

Natural products such as vinegar, apart from their common use as food condiments, have also been used for centuries as household disinfectants, sanitizers, and food preservatives to inhibit the growth of spoilage and pathogenic microorganisms [8,9,64,65]. The strong antimicrobial activity of vinegars is mainly associated with their high acetic acid content, in combination with other factors such as temperature, pH, and ionic strength. The presence of other compounds, which are renowned for their antimicrobial properties, such as polyphenols, lactic acid and other organic acids (C3-C6 fatty acids, 3-methyl butyric, succinic, tartaric, and citric acids, etc.), melanoidins (high molecular weight), ligustrazine, etc., also affects the antimicrobial potential of vinegars [22,44,66,67]. All the above classes of antimicrobial compounds are found in cereal vinegars, although compounds such as polyphenols are more abundant in wine and fruit vinegars [22,68].

Two main mechanisms have been associated with the antimicrobial activity of organic acids: the cytoplasmic acidification from proton release, which inhibits cellular functions such as energy production and regulation, and the intracellular accumulation of dissociated acid anions that inhibits cellular functions through several mechanisms [8,69]. For example, acetate accumulation has been shown to inhibit cell growth and methionine biosynthesis in *Escherichia coli*, due to both a reduction in intracellular methionine and accumulation of homocysteine, which is a toxic intermediate of methionine [69]. Other theories suggest that the antimicrobial action of organic acids is due to the uncoupling of electron transport from oxidative respiration, enzyme inhibition, modification of membrane permeability, inhibition of protein and DNA/RNA synthesis, etc. [70].

The main acids responsible for the antimicrobial activity of vinegars are acetic acid and D-gluconic acid produced by acetic acid bacteria [71,72], and lactic acid, citric acid, and 3-phenyllactic acid produced by lactic acid bacteria [20]. However, acetic acid and lactic acid are the organic acids that are more abundant in cereal vinegars [20,73]. Moreover, the processing conditions can enhance the acetic acid concentration of cereal vinegars, as demonstrated for traditional solid-state fermentation during SAV production [4].

Polyphenols also present antimicrobial activities, additionally to their strong antioxidant properties. The antibacterial activity of polyphenols has been attributed to several mechanisms, including alterations in cell membrane permeability, enzyme activity, pH, energy metabolism, DNA and protein synthesis, and other intracellular functions. These alterations may be due to polyphenol–protein complexation or H-binding and other interactions with the cell components [66]. Gallic acid, catechin, and vanillic acid are the major phenolic compounds found in cereal vinegars [17]. The antimicrobial potential of these compounds against various pathogens, such as *E. coli*, *Staphylococcus aureus*, *Pseudomonas aeruginosa*, *Enterobacter* spp., *Listeria* spp., *Mannheimia haemolytica*, *Pasteurella multocida*, etc., has been demonstrated in several studies [74,75,76,77,78,79].

γ-oryzanol from rice bran has also been found to exhibit antimicrobial activity against various bacterial and fungal strains, such as *E. coli*, *P. aeruginosa*, and *Fusarium graminearum* [12,13,80]. The in vitro antibacterial activity and AC of rice bran water/ethanol extracts against *Listeria innocua* and *E. coli* was also recently demonstrated [10].

Several scientific studies also dealt with the determination of the antimicrobial properties of vinegars against spoilage and pathogenic microorganisms (bacteria, yeast and fungi), aiming to develop effective and safe post-harvest treatments, to find alternative means of fighting antibiotic resistant strains, to replace synthetic preservatives and disinfectants, and to develop novel techniques such as edible coatings and nanoemulsions for both food and health care applications [8].

For example, the antimicrobial effect of RV on the survival of *E. coli*, *Salmonella enterica* serovars *Typhimurium*, *P. aeruginosa*, and *S. aureus* has been demonstrated in vitro [36,68,81]. RV was more effective against *S. aureus* compared to other vinegars [36]. However, the stronger antibacterial activity of pomegranate, apple, and grape vinegars could be attributed to the presence of sodium metabisulfite, which is a common antioxidant and antimicrobial additive in these products.

In order to find alternative ways to deal with the increasing incidents of nosocomial infections caused by antibiotic resistant strains, their susceptibility to acetic acid prepared from apple cider, molasses, dates, grapes, and grains was recently evaluated using conventional standard microbiological techniques. Specifically, strains were isolated from clinical samples of patients with nosocomial infections, with *P. aeruginosa* ATCC 27853 being the control strain. The results show that none of the clinical *P. aeruginosa* isolates were resistant to the tested organic ethanoic acids [82]. The antimicrobial capacity of fermented dark RV from unpolished rice against various human pathogens (bacteria and yeasts) was also assessed in vitro [59]. The vinegar exhibited strong antimicrobial activity against all species (*S. aureus*, *E. coli*, *Listeria monocytogenes*, *P. aeruginosa*, *S. typhimurium*, *Yersinia enterocolitica*, and *Lodderomyces elongisporus*), and it was higher than that of commercial antibiotics (carbenicillin 50 μg/mL and tetracycline 50 μg/mL).

Various cereal by-products and residues have been proposed as raw materials for vinegar production, and the antimicrobial properties of these products have also been assessed. For example, acetification of dried baby corn silks, an underutilized cereal by-product, led to 62–75 g/L acetic acid content during nine cycles of semi-continuous processing. A significant reduction in bioactive compounds (TPC and AC) was observed in both wine and vinegar, compared to the raw material, while among volatile compounds with antimicrobial properties five were found in both wine and vinegar (ethyl acetate, isoamyl acetate, isoamyl alcohol, hexanoic acid, octanoic acid) [83]. The results from an in vitro study on the inhibition of *E. coli* and *S. aureus* by the dried baby corn silks vinegar show that it was enhanced when compared with pure acetic acid [83].

Finally, the effect of commercially available RV on in situ pellicle formation and existing 24 h biofilms was evaluated [84]. The in situ biofilm formation took place on bovine enamel slabs mounted in individual splints and exposed intraorally for 3 min and 24 h, respectively. After rinsing with vinegar, the samples were analyzed via fluorescence, scanning electron, and transmission electron microscopy techniques, against samples rinsed with water as controls. It was found that vinegar can inhibit the formation of oral biofilm in situ, causing destruction of the pellicle. Specifically, vinegar rinsing reduced the outer globular layer of the pellicle, resulted in subsurface pellicle formation, reduced bacterial viability, and disrupted the 24 h biofilm. Therefore, it was concluded that rinsing with RV for only 5 s could alter the pellicle layer and significantly reduce the viability of planktonic microbes in saliva, decreasing biofilm formation [84].

### 2.3. Antidiabetic Properties

Diabetes is a chronic metabolic disease characterized by insulin resistance or a relative deficiency of insulin secretion, leading to increased blood glucose levels. Insulin is a hormone produced by pancreatic β-cells (known as the islets of Langerhans) and plays a key role in the metabolism of carbohydrates, fats, and proteins, and in the regulation of blood sugar. It allows the body to utilize the glucose produced from the above diet sources or to store it for future use. Therefore, it helps regulate the glucose levels in order to avoid hypo- or hyperglycemia incidents. There are two main types of diabetes, where most cases can be categorized based on their etiopathogenetic characteristics. Type 1 diabetes is characterized by a lack of insulin production, while Type 2 diabetes is characterized by ineffective use of insulin in the body, which causes the blood glucose concentration to increase. Many patients choose to either reduce the carbohydrate intake or to consume low glycemic index (GI) food. However, for the control of diabetes, postprandial glycemia (occurring after a meal consumption) is the most important parameter, since its insufficient control can lead to serious long-term complications including hypertension, cardiovascular disorders, blindness, and renal failure [85]. Therefore, there is a growing demand of consumers for foods that can help regulate blood glucose or even reduce postprandial glycemia. Vinegar has been shown to be one such type of food that can reduce the dietary glycemic load [86,87].

The antidiabetic effects and improvement of insulin sensitivity by vinegar have been demonstrated in both human and animal subjects, and several mechanisms for these effects have been proposed [88,89]. One suggestion is that vinegar and especially acetic acid may enhance the peripheral glucose uptake and its conversion to glycogen. Acetic acid, which is a major component of vinegars, improves glucose metabolism in rats by activating gluconeogenesis and inactivating glycolysis through the suppression of xylulose-5-phosphate accumulation and inactivation of fructose-2,6-bisphosphate synthesis in the liver, and phosphofructokinase-1 activity in the skeletal muscle [90,91]. In addition, human studies have supported the previous hypothesis that the glycemic benefits of vinegar may be attributed to an improvement in glucose uptake [92,93]. Vinegar consumption in combination with exercise training may improve glucose uptake and increase the expression of genes involved in mitochondrial respiration and fatty acid oxidation. Vinegar acetic acid may stimulate the adenosine 3′,5′-monophosphate activated protein kinase (AMPK), which optimizes the muscle glycogen replacement and enhances glucose uptake after exercise [94]. Other suggested mechanisms include interference with the enzymatic digestion of complex carbohydrates [95], delayed gastric emptying [96], alteration of the glycolysis/gluconeogenesis cycle in the liver [97], and enhancement of satiety [86].

Cereal vinegars present an inhibition effect on α-glucosidase, and therefore may be used to prevent or medically treat type II diabetes by reducing the absorption of dietary carbohydrates. Fan et al. [23] studied Chinese cereal SAVs (SoV, OaV and TBV) and ZAVs (Zhenjiang Hengshun vinegar, ZHV, and Liubiju rice vinegar, LRV) and determined an in vitro inhibitory activity over α-glucosidase. More specifically, SoV presented the highest inhibition followed by OaV and TBV. The higher inhibition activity of SAVs compared to ZAVs was attributed to their higher content of phenolic and alkaloid components. It is known that phenolic compounds (flavonoids, phenolic acids, tannins) and alkaloids from fruits and vegetables inhibit α-glucosidase, which may be attributed to complex formation with these compounds, driven mainly by non-covalent interactions (i.e., van der Waals forces, H-bonding). However, the specific mechanism of action of these compounds against α-glucosidase is not clear and more studies are required to clarify that both in vitro and in vivo [98,99,100].

Τhe potential antidiabetic properties of cereal vinegars have also been proved in vivo. For example, the oral administration of 2 mL/kg/day for 1 month of white RV to streptozotocin induced diabetic rats, resulted in lower body weight loss, lower fasting and random blood glucose levels, higher fasting serum insulin levels, and higher β-cell proportion [101]. In a similar study, the oral intake of TBV (for 1 month) reduced blood glucose levels up to 17% [102].

More recently, a traditional ZAV extract (100–400 μg/mL), rich in polyphenols, was found able to increase glucose uptake and consumption, enhance glycogen synthesis and attenuate gluconeogenesis in high glucose-induced insulin resistant HepG2 (IRHepG2) human liver cancer cells. In addition, it improved the insulin resistance by inhibiting the expression of the phosphorylated insulin receptor substrate-1 (IRS-1), and by activating the phosphatidylinositol 3-kinase (PI3K)/protein kinase B (PKB or Akt) pathway in IR-HepG2 cells. Moreover, ZAV reduced the generation of ROS and the expression of phosphorylated c-Jun NH terminal kinase (JNK) in the IR-HepG2 cells [30].

Finally, a systematic review and meta-analysis of the effectiveness of vinegar consumption in improving glycemic control in type 2 diabetes mellitus patients, from relevant studies, showed that vinegar can significantly improve fasting blood glucose levels and hemoglobin A1c (HbA1c) levels and reduce the total cholesterol and low-density lipoprotein postintervention. It was concluded that vinegar could be incorporated into the diet of diabetic patients [103].

### 2.4. Regulation of Lipid Metabolism and Antiobesity Properties

Studies regarding the antilipidemic effect of vinegar are more recent and primarily focused in animal-based experiments, while only limited results from human trials are available. These studies have shown that chronic administration of a specified amount of acetic acid or vinegar can significantly reduce the concentration of total cholesterol, triglycerides, and low-density lipoprotein cholesterol (LDL-c), and increase the concentration of high-density lipoprotein cholesterol (HDL-c) [90,103,104,105].

There are several potential mechanisms proposed for the regulation of lipid metabolism by vinegar and acetic acid. The first is the inhibition of cholesterol formation and lipogenesis in liver by acetic acid and vinegar through the activation of the AMPK pathway, as several in vivo studies have proposed [15,90,106]. Acetic acid is a building block in the synthesis of acetyl-coenzyme A (acetyl-CoA), during which adenosine triphosphate (ATP) is consumed, and adenosine monophosphate (AMP) is produced. The increase in the AMP/ATP ratio activates the AMPK pathway, which interferes with glucose uptake and free fatty acid oxidation in the skeletal muscle and inhibits gluconeogenesis, glycolysis, lipogenesis, and cholesterol formation in the liver leading to decreased blood glucose and lipid levels, and increased insulin sensitivity [107]. A second proposed mechanism involves the increase in the expression of acetyl-CoA oxidase by acetic acid and vinegar, and therefore leading to suppression of plasma triglycerides [90,106]. Acetic acid also leads to a reduction in blood lipid content through the promotion of fatty acid oxygenolysis, and fecal excretion of bile acid by stimulation of secretin release [90]. Vinegar also plays a beneficial role in affecting the HDL-c and LDL-c levels, which is associated with the reduction in the glycemic index of foods that contain acetic acid, as well as the vinegar polyphenols and flavonoids that decrease the LDL-c levels [108,109].

Vinegar consumption has also been associated with antiobesity effects and body weight regulation. At least five different mechanisms have been proposed to explain the effect of vinegar on weight loss [104,110]: (1) decrease in lipid synthesis (attenuation of several lipogenic genes in the liver that are required for the conversion of glucose to fatty acids), (2) increase in lipolysis (increased expression of several lipolytic genes that encode fatty oxidation enzymes), (3) increase in energy consumption, (4) increase in oxygen consumption (through myoglobin that facilitates oxygen diffusion), and (5) increase in postprandial satiety and decrease in energy intake (reduction in food glycemic index and stabilization of postprandial blood glucose levels). Thus, the ability to promote fatty acid oxidation, enhance lipolysis, inhibit lipogenesis and regulate the levels of HDL-c and LDL-c suggests that vinegar can be used as a natural complementary antiobesity medication [104,110,111]. However, more intense, long-term clinical trials are necessary in order to evaluate and prove the effects of vinegar on lipid metabolism and obesity.

Several studies have demonstrated that consumption of cereal vinegars ameliorates obesity-related conditions in humans [112,113,114,115] and experimental animals [116,117,118,119]. For example, a 12-week, randomized, double-blind, placebo-controlled trial demonstrated a significant decrease in body weight and a possible decrease in body mass index in obese adults who consumed KuV (870 mg/day) [112]. In a similar study, where the same intake of KuV was combined with 10 min daily exercise for 12 weeks, fat-derived energy consumption increased, while suppressing the carbohydrate-derived energy consumption. Participants with the largest amount of body fat presented the highest decrease in fat due to KuV action [113]. An open-label, non-controlled study with oral administration of KuV concentrate (1000 mg/day) for 8 weeks also showed a reduction in visceral fat and blood pressure and an increase in HDL-c levels [115]. Likewise, in a randomized, double-blind, placebo-controlled intervention study that combined intake of KuV and garlic (200 mg/day for 12 weeks), an improvement in total cholesterol and LDL-c levels was revealed [114].

In rats, the oral administration of KuV concentrate (100 mg/kg body weight for 8 weeks) decreased the adipocyte size via inhibition of dietary fat absorption and reduction in PPARγ and the fatty acid transport adipocyte protein 2 (aP2) mRNA expression in adipocytes (fat cells) [117]. In another study on rats, the oral intake of maize vinegar (0.3 mL/day for 1 month) resulted in reduced serum total cholesterol, triglycerides, total weight of fat around the genitalia, serum HDL, and atherosclerosis index [116]. In mice fed with a high-fat diet, the administration of KuV concentrate resulted in significantly lower body weight [119]. The administration of 1% of freeze-dried SAV powder for 35 days to high-fat diet-fed mice resulted in a significant reduction in serum triglycerides, total cholesterol, and serum LDL-c levels, while the serum HDL-c was significantly increased [118]. Black vinegar powder supplementation also had a lipid-lowering effect in hamsters fed with a high-fat-cholesterol diet, while its antiobesity effects under a chronic high-fat condensed diet were also shown [15]. Finally, in a recent study by Zhao et al. [120], it was shown that vinegar as a whole, and not acetic acid, is effective in reducing plasma total cholesterol and non-HDL-c concentrations, at least in hypercholesterolemic hamsters fed a high-cholesterol (0.2%) diet. The study evaluated the effect of the administration of balsamic vinegar, SAV, and pure acetic acid solutions (20 mg/mL), at 8 mL solution/kg body weight, for 9 weeks. Real-time PCR analysis showed that the vinegars significantly up-regulated the mRNA of cholesterol 7 α-hydroxylase (CYP7A1) in the liver.

Acetic acid, as with all short chain fatty acids (SCFAs), can be absorbed by the colonic epithelial. The results of Ruppin et al. [121] indicate the existence of two mechanisms for colonic SCFA absorption. The 1st mechanism, which accounts for about 60% of the total SCFA absorption, comprises a nonionic diffusion of protonated SCFA that involves the consumption of luminal CO_2_. The second mechanism is the cellular uptake by ionic diffusion of the Na or K salt of the SCFAs. The mechanism for this transport was described well in a recent study [122]. In brief, SCFAs require transporters in order to be absorbed since they are present in their ionized form in the colonic pH (pKa < colonic pH). These transporters are expressed at different levels in the small intestine and colon, and cellular uptake of SCFAs in their anionic form occurs through H^+^- or Na^+^-coupled transporters.

Recently, a novel oligoglucan-melanoidin complex that was isolated from KuV was found to suppress adipogenesis in vitro [40]. Isolation was carried out by organic solvent extraction, followed by size-exclusion chromatography. The melanoidin complex, at ~109 μg/mL, significantly suppressed adipogenesis that was determined as inhibition of cytoplasmic droplet accumulation in 3T3-L1 murine fibroblasts (preadipocytes) that had been induced for adipocyte differentiation. This activity was considered in part responsible for the antiobesity effects of KuV that was observed in various human and animal studies [40].

Ligustrazine is another bioactive compound found in Chinese black vinegar. Chen et al. [44] investigated the potential effects of this compound on intracellular cholesterol modulation in HepG2 cells (an immortalized human liver carcinoma cell line). The results demonstrate that ligustrazine (35, 40, 45, and 50 μg/mL) could induce intracellular cholesterol efflux and improve the endothelial function through inhibiting ROS and increasing the antioxidant enzymes superoxide dismutase and catalase. It also increased the liver X receptor and PPARγ gene expression in HepG2 cells. Protein expression of ATP-binding cassette transporter 1 was also upregulated in a dose-dependent manner. Based on these results, it was concluded that ligustrazine has hypolipidemic effects via the modulation of intracellular cholesterol efflux, ROS inhibition, increased antioxidant enzyme activities, and direct regulation of PPAR and LXR gene expression (elevation of the PPARγ-LXRα-ABCA1 pathway) [44].

### 2.5. Anticancer Properties

Cancer is a disease characterized by uncontrolled proliferation of malignant cells, which then attack nearby tissues. The available evidence from medical and nutritional sciences shows that diet, and especially fermented food, plays a crucial role in the regulation of several diseases, including cancer [123]. Reactive species such as free radicals (superoxide radicals, hydroxyl radicals, etc.), when overproduced in an organism, may cause damage to normal cellular functions and ultimately cancer [124]. The AC of vinegar is evident as described above, and therefore, its potential anticancer effects through this mechanism are well established. However, other possible mechanisms for the anticancer activity have been proposed.

Until now, only a few studies involving animal subjects and cultured cells, focusing on the anticancer activities of vinegars, have been published, the majority of which are related to cereal vinegars. For example, ethyl acetate extracts of SAV and KuV significantly inhibited the proliferation of cancer cells in vitro [55,125]. More specifically, cancer cell lines such as A549 (lung), Hep-G2 (liver), MDA-MDB-231 (breast), Hela (cervical), and HCT116 (colon) were studied in the case of SAV. Polar fractions were isolated from the ethyl acetate extract of SAV by preparative chromatography and the inhibition ratios of 100 mg extract/mL were found to be higher in the case of lung cancer cells (61.5%), followed by liver (49.1%), breast (44.4%), and cervical cancer cell lines (33.5%). No inhibition effect was reported on colon cancer cells. In addition, the IC50 on lung cancer cells was 18.3 μg/mL, which was significantly lower than the corresponding effect on normal cells (281 μg/mL), confirming the promising anticancer activity of this extract, especially against lung cancer [125]. In a similar study, the ethyl acetate extract of KuV from unpolished rice, on the proliferation of a variety of human cancer cell lines such as colon adenocarcinoma (Caco-2), lung carcinoma (A549), breast adenocarcinoma (MCF-7), bladder carcinoma (5637), and prostate carcinoma (LNCaP), were investigated using the Alamar Blue assay. The extract inhibited the proliferation of all tested cell lines in a dose-dependent manner (0.025–0.1%), and the most promising results were obtained in the case of Caco-2 cells (up to 62% inhibition at 0.025% dose level). The inhibitory effect of KuV extract on the growth of human cancer cell lines was attributed to the induction of cell cycle arrest and apoptosis [55]. The novel Izumi version of KuV, made from unpolished rice as described above, was also found to inhibit (at 10 μL/mL) the proliferation of human skin squamous cell carcinoma cells (HSC-5) [126]. The inhibition was higher compared to that of traditional grain vinegar, but apoptosis was not induced. However, an increase in the cellular levels of RIPK3 (receptor interacting serine/threonine protein kinase 3) and HMGB1 (high mobility group box 1) proteins was reported, indicating that Izumi inhibits the proliferation via programmed necrosis or necroptosis [126]. Budak et al. [9] also stated that the hindering of cell proliferation can be aided by KuV, thus it can be used as a complementary treatment for various cancer cells.

In the case of in vivo studies, several mouse and rat animal models have been used to evaluate the potential anticancer activity of cereal vinegars and especially RVs. In a study using mice with Sarcoma 180 and Colon 38 tumor cells, the incorporation in their diet of 0.5% post-distillation slurry vinegar of rice Shochu (a Japanese distilled beverage), for 72 days, significantly decreased the sizes of tumors and prolonged the lifespan of mice [127]. On the other hand, the administration of 0.32% of 10-fold concentrated KuV in the diet of cancer cell–transplanted mice had no effect on the tumor size. However, the addition of 2% Kurosu “moromimatsu” powder (the insoluble sediment that remains after the KuV fermentation, which is rich in dietary fiber and peptides) prolonged the lifespan of cancer cell-transplanted mice, inhibited tumor progression, and reduced nitrotyrosine production and activation of matrix metalloproteinases (MMP), but did not induce apoptosis [128]. In a similar study, the addition of Kurosu moromimatsu (2%) in the diet of diethylnitrosamine-induced hepatocellular carcinoma rats, prolonged their survival and inhibited the growth of cancer [129]. The effects of incorporating an ethyl acetate KuV extract in drinking water on the development of azoxymethane-induced colon carcinogenesis, were investigated in a rat model by Shimoji et al. [53]. The results show that water containing 0.05% of ethyl acetate KuV extract for 35 weeks significantly inhibited azoxymethane-induced colon cancer in rats [53]. The antitumor properties of KuV and Kurozu moromimatsu, along with other health benefits (improvement of dyslipidemia, prevention of hyperglycemia, antiallergic activity, etc.), were recently reviewed by Shibayama et al. [54].

The antitumor factors in vinegar and corresponding mechanisms have not been fully identified; however, they are mostly linked to the high phenolic content of vinegars, and as a result, their high AC. Therefore, more research is needed in order to determine the specific mechanisms of the anticancer activity of vinegars. Furthermore, future studies should also focus on possible interactive effects among vinegar compounds in producing these anticancer effects. The epidemiologic studies are also limited and provide controversial results. For example, case-control studies conducted in China demonstrated that vinegar ingestion was associated with a decreased risk for esophageal [130] and non-cardia stomach cancer [131], but vinegar ingestion was also associated with a 4.4-fold greater risk of bladder cancer in a case-control study in Serbia [132]. Therefore, a case-by-case evaluation is necessary to assess the anticancer activity of cereal vinegars.

### 2.6. Antihypertensive Properties

Hypertension is a major public health problem and an important risk factor for cardiovascular diseases such as coronary artery disease, stroke, heart failure, atrial fibrillation, peripheral vascular disease, chronic kidney disease, and atherosclerosis [133]. Both hereditary and environmental factors have been associated with hypertension. Certain food components, such as natural polyphenols, and foods including vinegar, have been found to have regulatory effects on blood pressure, although scientific evidence is limited [134].

In several studies on animal subjects, the angiotensin-converting enzyme (ACE) was found to be inhibited by vinegar. For example, RV was found to be able to inhibit ACE activity and reduce blood pressure in vivo [135,136]. The possible antihypertensive effects of RV were evaluated during an 8-week vinegar administration (diet with 6% *w*/*w* vinegar) in the diet of spontaneously hypertensive rats. The results show a significant reduction in blood pressure and renin activity compared to the control rats. This effect was attributed to the significant reduction in renin activity and the subsequent decrease in angiotensin II [135]. Furthermore, the combination of RV with ginger extract [137] or *Undaria pinnatifida* sporophyll [138] in the diet of hypertensive rats also resulted in a reduction in blood pressure.

Na et al. [134] investigated whether RV exerts an antihypertensive effect by activating the AMPK pathway in spontaneously hypertensive rats. The results led to the conclusion that vinegar (7 mL/kg body weight of spontaneously hypertensive rats) activates AMPK by increasing the AMP/ATP ratios, thereby it increases the expression of PPARγ coactivator 1 alpha (PGC-1α) and PPARγ and inhibits the expression of angiotensin II receptor type 1 (AT1R). Acetic acid was considered responsible for these antihypertensive effects of vinegar.

Evidence from human studies is also available regarding the antihypertensive activities of cereal vinegars. For example, in a double-blind, placebo-controlled, randomized study, involving volunteers with normal, mild, or moderately high blood pressure, a positive effect from the consumption of fermented drinking water (90 mL daily) containing RV was reported. After 12 weeks of administration, followed by 4 weeks of no intake (total 16 weeks), a significantly decreased systolic (up to −7.6 ± 4.0 mm Hg) and diastolic (up to −10.6 ± 4.0 mm Hg) blood pressure compared to the beginning of the study was observed. In addition, no abnormal changes in hematological or blood chemistry variables, urinalysis, heart rate, or body weight were recorded in the study groups [139]. Additionally, the intake of concentrated KuV by healthy adults resulted in significantly lower blood pressure [140].

The antihypertensive properties of cereal vinegars may also be attributed to their GABA content. GABA is considered a potent bioactive compound, due to its numerous health benefits and its hypotensive effect that has been demonstrated in animals and in human intervention trials [141]. Indeed, a single oral administration (0.5 mg/kg) significantly lowered the systolic blood pressure in spontaneously hypertensive rats, suggesting that GABA has an antihypertensive effect due to its inhibition of noradrenaline release from sympathetic nerves in the mesenteric arterial bed via presynaptic GABA_B_ receptors [142]. A human study revealed that a daily supplementation of 80 mg of GABA reduces blood pressure in adults with mild hypertension [143]. The content of GABA in cereal vinegar is relatively low in order to fulfill the demand for such high supplementation. Therefore, studies on the conditions to brew RV with high GABA content (up to 100 mg/L) were carried out using sprouted rice as raw material [144]. Nevertheless, cereal vinegars can be considered a source of GABA, and therefore their antihypertensive effect may also be partially attributed to this compound.

### 2.7. Effect on Neurodegenerative Diseases

The most common neurodegenerative diseases are Alzheimer’s and Parkinson’s disease, which affect millions of people worldwide. They occur when nerve cells in the brain or peripheral nervous system lose function over time and ultimately die. Neurodegenerative diseases belong to free radical-induced diseases, and therefore several compounds occurring in cereal vinegars like polyphenols, flavonoids and melanoidins may play a pivotal role in their prevention [145]. As was also highlighted in this review, cereal vinegars possess high AC, and therefore may positively affect these diseases. In addition, the epidemiology of neurodegenerative diseases has linked them with other diseases, such as obesity, hypertension, diabetes, cardiovascular disease, etc. [146], on which cereal vinegars have been shown to have positive effects. However, targeted research studies regarding the mechanism of this effect are limited and mainly focus on apple cider vinegar [147,148]. For example, concentrated KuV or Kurosu moromimatsu were evaluated regarding their potential in ameliorating cognitive dysfunction in the senescence-accelerated P8 mice [149]. The mice were fed 0.25% (*w*/*w*) concentrated KuV or 0.5% Kurozu moromimatsu for 4 or 25 weeks. KuV was found to suppress cognitive dysfunction and amyloid accumulation in the brain, while the effect of Kurozu moromimatsu was not significant. Finally, intriguing results were recently reported about the production of fluorescent nanoparticles (FNPs) during Chinese mature vinegar fermentation. The FNPs interacted with dopamine, a well-known neurotransmitter, which may indicate potential health implications of foodborne nanoparticles [150]. Although research regarding the effect of cereal vinegars, and food in general, on neurodegenerative diseases is limited, this field is very attractive, and in-depth in vitro and in vivo studies are required to define the underlying mechanisms.

### 2.8. Vinegar Side Effects

Despite their various health benefits, vinegar may also have some adverse effects. In general, vinegar ingestion at mealtime is gaining popularity; however, vinegar is highly acidic with a pH value near to, and in some cases even lower than 3. Frequent consumption may result in dental erosion (irreversible loss of dental hard tissue; enamel and dentin) [151]. In vitro studies revealed this effect [152,153]. Furthermore, in a recent study, the effect of daily vinegar ingestion during an 8-week randomized trial was examined [154]. The participants consumed vinegar twice a day (2 tablespoons vinegar in a cup of water; 3.6 g acetic acid) or a commercial vinegar pill (control; 0.045 g acetic acid) at mealtime. The basic erosive wear examination (BEWE) scores did not change in the control group (since the pill was quickly swallowed and the oral cavity was not exposed to the high acidity), but increased by 18% in the vinegar group, suggesting the potential of vinegar to erode teeth.

Vinegar has also been associated with some cases of esophageal injury. In Hong Kong, a 39-year-old woman consumed 1 tablespoon of white RV in the belief that it would dissolve the crab shell stuck in her throat [155]. However, endoscopy revealed inflammation of the oropharynx and second-degree caustic injury of the esophagus extending to the cardia. Chronic intake of excessive amounts of vinegar can also cause similar implications. In Korea, a 15-year-old boy consumed daily for 1 month 100–150 mL of an undiluted pomegranate-fermented vinegar beverage (pH 2.6), despite the fact that the manufacturer recommended a 3:1 water:vinegar dilution [156]. The endoscopic examination revealed multiple longitudinal ulcers, concurrent mucosal hemorrhage, and denuded mucosa in the whole of the esophagus. In Austria, a 28-year-old woman presented high urinary excretion of K, Na, and bicarbonate, and stimulated plasma renin activity after a daily consumption of 250 mL of vinegar (12.5 g acetic acid) diluted in water and as salad dressing for 6 years [157]. In another case, a 72-year-old Caucasian man added 2 tablespoons daily of household vinegar to his diet for almost 2 weeks, resulting in hospital with intractable singultus (hiccups) for approximately 9 days, associated with anorexia and epigastric pain [158].

In addition, vinegar has been associated with several burns due to traditional medicine remedies. Some examples include a split-thickness leg burn after applying a self-concocted mixture consisting of white vinegar and aspirin [159], chemical burns after following an Internet-based protocol for nevi removal using apple cider vinegar [160], and face chemical burns after applying a vinegar-containing solution in an effort to alleviate pediculosis capitis [161]. To conclude, despite the numerous health benefits of vinegars, chronic intake of excessive amounts and inappropriate use may cause serious health problems.

## 3. Conclusions

A variety of bioactive components such as organic acids, natural phenolics, vitamins, minerals, etc., have been associated with the health properties of cereal vinegars. Acetic acid is the major component in vinegars that is responsible for the strong antibacterial properties as well as for the regulation of postprandial blood sugar levels, regulation of lipid metabolism, antiobesity, and antihypertensive effects of vinegars. Phenolic compounds, on the other hand, are mainly responsible for the antioxidant properties of cereal vinegars, and the related oxidative stress reduction, anticancer, antidiabetic, hepatoprotective, and hypolipidemic effects of vinegar consumption. Among other bioactive components found in cereals and cereal vinegars, specific components such as γ-oryzanol, ligustrazine, GABA, alkaloids, melanoidins, etc., have gained increased attention due to their strong bioactive properties, including antioxidant, anti-inflammatory, antimicrobial, anticarcinogenic, immunoregulatory, and other effects. These compounds derive either from the raw material (e.g., alkaloids, γ-oryzanol and fraglide-1), or are produced during the fermentation, aging, and other processes involved in cereal vinegar production (e.g., GABA, ligustrazine and melanoidins) Therefore, both the raw material (including the cereal bran, husk, and chaff) and the vinegar manufacturing process (e.g., submerged or solid state fermentation, thermal treatment, aging process and duration, etc.) are essential for optimum enrichment of the final products in bioactive components that can confer positive health effects to consumers.

To conclude, this literature review highlights that the inclusion of cereal vinegars in the diet (as dressing or acidifier) can contribute to the health status and integrate current therapies against various pathologies. However, as pointed out in a systematic review and meta-analysis [103], which acknowledges the health benefits of vinegars and proposes vinegar consumption as part of a healthy diet, the amount of vinegar used to establish its bioactive properties varies across studies and is relatively small. Therefore, special attention should be paid to the generalization of the outcomes of clinical studies [103]. In addition, considering that the available data are relatively limited, further research combined with epidemiologic and case studies are essential for the scientific substantiation of the health benefits of cereal vinegars, that may also allow specific health claims from vinegar manufacturers and better consumer understanding.

## Figures and Tables

**Table 1:** Bioactive components in cereal vinegars.

Compounds	Reported Levels in Cereal Vinegars	Potential Bioactivity/Functionality	Types of Assayed Vinegars
**Organic acids**			
Acetic acid (AA)	AA and LA prevail in cereal vinegars (>70–80%) [16,17]. Mean AA levels (g/L) in Chinese cereal vinegars 22.5–71.5 g/L (38.8–83.6% of total acids) [16], in SAVs (SBB) 12.6–33.0 [17], in ZAV (glutinous rice/bran) 17.7 [17], in 0–7-year-old traditional ZAVs 46.8 [18], in 0–7-year-old industrial ZAVs 39.1 [18], and in 10-year-old ZAV 66.1 [19].	Acidity and flavor in vinegars. Antimicrobial properties. Regulation of lipid metabolism. Regulation of postprandial blood sugar levels.	RV, SAV cereal vinegars (SoV, OaV, TBV), ZAV vinegars (ZHV, LRV, traditional, industrial, aged and non-aged), WBrV, TDMV, FMV, SBrV [9,16,17,18,19,20,21,22,23,24].
Lactic acid (LA)	Mean LA levels (g/L) in Chinese cereal vinegars 4.2–50.1 (11.2–40.4% of total acids) [16], in SAVs (SBB) 11.0–16.3 [17], in ZAV (glutinous rice/bran) 15.2 [17], in 0–7-year-old traditional ZAVs 24.1 [18] in 0–7-year-old industrial ZAVs 26.8 [18], in 10-year-old ZAV 32.8 [19], and in traditional Chinese rose vinegar (sticky rice) 1.33 [20].		
L-pyroglutamic acid (L-P)	Mean L-P content (g/L) in 0–7-year-old traditional ZAVs 16.4 [18], in 0–7-year-old industrial ZAVs 13.7 [18], and in 10-year-old ZAV 13.6 [19].		
Oxalic acid (OA)	Mean OA content (g/L) in 0–7-year-old traditional ZAVs 1.7 [18], in 0–7-year-old industrial ZAVs 1.4 [18], and in 10-year-old ZAV 1.9 [19].		
Tartaric acid (TA)	Mean TA content (g/L) in SAVs (SBB) 2.4–3.6 [17], in ZAV (glutinous rice/bran) 3.8 [17], in 0–7-year-old traditional ZAVs 0.9 [18], in 0–7-year-old industrial ZAVs 0.8 [18], and in 10-year-old ZAV 0.9 [19].		
Succinic acid (SA)	Mean SA content (g/L) in SAVs (SBB) 0.5–0.7 [17], in ZAV (glutinous rice/bran) 0.2 [17], in 0–7-year-old traditional ZAVs 9.2 [18], in 0–7-year-old industrial ZAVs 3.1 [18], and in 10-year-old ZAV 5.3 [19].		
Malic acid (MA)	Mean MA content (g/L) in SAVs (SBB) 0.2–0.8 [17], in ZAV (glutinous rice/bran) 1.0 [17], in 0–7-year-old traditional ZAVs 2.0 [18], in 0–7-year-old industrial ZAVs 1.8 [18], and in 10-year-old ZAV 2.2 [19].		
Citric acid (CA)	Mean CA content (g/L) in SAVs (SBB) 0.4–1.0, and in ZAV (glutinous rice/bran) 0.4 [17].		
Propanedioic acid (PA)	Mean PA content (g/L) in SAVs (SBB) 1.7–2.9, and in ZAV (glutinous rice/bran) 0.6 [17].		
Quinic acid (QA)	Mean QA content (g/L) in SAVs (SBB) 3.7–5.7, and in ZAV (glutinous rice/bran) 6.0 [17].		
Other acids at lower levels: butyric, caproic, formic, fumaric, glycolic, D-gluconic, hexanoic, 3-hydroxypropanoic and other hydroxy acids, α-ketoglutaric, 2-methylpropanoic, 3-methylbutanoic, octanoic, pentanoic, propanoic, higher MW fatty acids, etc.	Total mean organic acid content (g/L) 75–176 (including acetic acid) in SAV vinegars [16,23], 85.3 in ZAVs [16], 101.1 in 0–7-year-old traditional ZAVs [18], 49.5–77.3 in 0–10-year-old ZAVs [19], 122.6 in SBrVs [16], 87.3 in FMVs [16], and 55.6 in TDMVs [16]. Total acidity in 1–10 years aged SAVs was in the range 35–80 g/L (generally acidity increases with aging time) [24].		
**Carbohydrates and sugar alcohols**			
Glucose (Glu), fructose (Fru), arabinose (Ara), xylose (Xyl)	Glu is the major sugar in SAVs (~38.7%), and Fru in other Chinese vinegars (>38.9%) (SBrVs, TDMVs, ZAVs), followed by Ara and Xyl [16]. Fru and Glu levels in Chinese cereal vinegars are found in the 1.6–13.9 and 2.7–8.7 g/L, respectively [16].	Fermentable sugars serve as C-sources for the vinegar fermentation microflora. Several polysaccharides exert anticoagulation, antioxidant, immunoregulatory, and prebiotic properties.	RV (brown, sticky rice), SoV, SAV, ZAV, TBV, TDMV, FMV, SBrV [16,20,22,25,26].
Other components at lower levels:allose, arabitol, deoxygalactose, dulcitol, erythritol, fucose, galactose, β-Glucans, glycerol, hemicellulosic arabinoxylans, glucose/mannose-rich polysaccharides, inositol, maltose, mannitol, mannose, microbial extracellular polysaccharides, pinitol, rhamnose, ribitol, ribose, sorbitol, threitol, trehalose, xylitol, etc.	Total sugar content (g/L) is in the range 2.6–28.7 in SAVs, and total mean content is 21.6 in SBrVs, 17.3 in TDMVs, and 17.2 in ZAVs [16]. Pentose levels (g/L) in ZAVs is in the range 1.2–3.8, and in SBrVs 2.2–2.6 [16]. Hexose levels (g/L) in ZAVs is in the range 6.8–23.4, and in SBrVs 11.2–21.0 [16].		
**Minerals**			
Potassium (K)	Mean K content in KuV 3067 mg/kg [26], and in SoV (Taiwan) 365 ± 16 ppm [27].	Essential nutrients. Blood pressure regulation. High bioavailability as vinegar components. Decreased absorption of dietary fat.	RV, KuV, SAV cereal vinegars (SoV, OaV, TBV), Taiwan SoV, ZAV vinegars (ZHV, LRV), KuV [23,27,28].
Sodium (Na)	Mean Na content in SoV (Taiwan) 72.6 ± 0.2 ppm [27].		
Calcium (Ca)	Mean Ca content (mg/kg) in KuV 3.1 [28], in SAVs 651–1246 (the highest levels in OaV) [23], in ZAVs 31–134 [23], and in SoV (Taiwan) 5.7 ppm [27].		
Phosphorus (P)	Mean P content (mg/kg) in SAVs 3335–4723 mg/kg (the highest levels in OaV), and in ZAVs 22–1968 [23].		
Magnesium (Mg)	Mean Mg content in KuV 953.7 mg/kg [28], and in SoV (Taiwan) 12.6 ppm [27].		
Iron (Fe)	Mean Fe content (mg/kg) in KuV 24.7 [28], in SAVs 140–305 (the highest levels in OaV) [23], in ZAVs 1.5–38 [16], and in SoV (Taiwan) 1.8 ppm [27].		
Manganese (Mn)	Mean Mn content (mg/kg) in KuV 32.9 [28], in SAVs 40–70 (the highest levels in OaV) [23], in ZAVs 0.04–45 [23], and in SoV (Taiwan) 0.8 ppm [27].		
Zinc (Zn)	Mean Zn content (mg/kg) in SAVs 18–38 (the highest levels in OaV) [23], in ZAVs 0.2–17 [23], and in SoV (Taiwan) 0.3 ppm [27].		
Cooper (Cu)	Mean Cu content in SoV (Taiwan) 0.03 ppm [27].		
Selenium (Se)	Mean Se content (mg/kg) in KuV 0.002 [28], in SAVs 0.01–0.02 (the highest levels in OaV) [23], and in ZAVs 0.001–0.012 [23].		
Iodine (I)	Mean I content (mg/kg) in SAVs 0.1–0.53 (the highest levels in OaV), and in ZAVs 0.07–0.31 [23].		
**Vitamins**			
Niacin, Nicotinamide (B3)	The B3 vitamers prevail in SAV and ZAV vinegars [23]. Total mean content (mg/100 g) in SAVs 29–38 niacin and 13–35 nicotinamide (the highest levels in OaV) [23]. Total mean content (mg/100 g) in ZAVs 2.3–12 niacin and 1.2–14 nicotinamide [23].	Essential nutrients. Important for energy metabolism. Lowering cholesterol excretion (B3). Good skin health and vision (B1). Brain function (B1, B3). Blood vessel expansion (B3).	SAV cereal vinegars (SoV, OaV, TBV), ZAV vinegars (ZHV, LRV) [22,23].
Riboflavin (B2)	Mean content (mg/100 g) of B2 in SAVs 0.9–1.6 (the highest levels in OaV) [6], and in ZAVs 0–2 [23].		
Thiamine (B1)	Mean content (μg/100 g) of B1 in SAVs 0.4–0.7 (the highest levels in OaV), and in ZAVs 0–0.13 [23].		
**Amino acids**			
Glutamic acid (Glu)	Mean content (mg/100 g) of Glu in SAVs 463–888 [23], in ZAVs 295–659 [23], and in dried KuV 3306 [28].	Regulation of cellular metabolism. Improvement of immunity. Promotion of brain development. Antioxidant activity. Regulation of glycemia levels. Antiobesity effects. Contribution to the product taste (umami, sweet, or bitter).	RV (glutinous, non-glutinous, black), SAV cereal vinegars (SoV, OaV, TBV), ZAV vinegars (ZHV, LRV, traditional, industrial, aged and non-aged), dried KuV, TDMV, FMV, SBrV [15,16,19,22,23,28]
Asparagine (Asn)	Mean content (mg/100 g) of Asn in SAVs 130–227, and in ZAVs 51–422 [23].		
Alanine (Ala)	Mean content (mg/100 g) of Ala in SAVs 119–251 [23], in ZAVs 153–170 [23], in 10-year-old ZAV 78 [19], and in dried KuV 979 [28].		
Valine (Val)	Mean content (mg/100 g) of Val in SAVs 107–216 [23], in ZAVs 106–118 [23], in 10-year-old ZAV 89 [19], and in dried KuV 654 [28].		
Proline (Pro)	Mean content (mg/100 g) of Pro in SAVs 130–294 [23], in ZAVs 96–180 [23], and in dried KuV 1185 [28].		
Glycine (Gly)	Mean content (mg/100 g) of Gly in SAVs 100–175 [23], in ZAVs 81–131 [23], in 10-year-old ZAV 144 [19], and in dried KuV 754 [28].		
Serine (Ser)	Mean content (mg/100 g) of Ser in SAVs 62–112 [23], in ZAVs 50–83 [23], and in dried KuV 530 [28].		
Isoleucine (Ile)	Mean content (mg/100 g) of Ile in SAVs 51–106 [23], in ZAVs 57–81 [23], in 10-year-old ZAV 67 [19], and in dried KuV 443 [28].		
Leucine (Leu)	Mean content (mg/100 g) of Leu in SAVs 93–175 [23], in ZAVs 96–98 [23], in 10-year-old ZAV 154 [19], and in dried KuV 734 [28].		
Methionine (Met)	Mean content (mg/100 g) of Met in SAVs 11–21 [23], in ZAVs 21–22 [23], in 10-year-old ZAV 17 [19], and in dried KuV 161 [28].		
Threonine (Thr)	Mean content (mg/100 g) of Thr in SAVs 50–92 [23], in ZAVs 51–137 [23], in 10-year-old ZAV 141 [19], and in dried KuV 451 [28].		
Histidine (His)	Mean content (mg/100 g) of His in SAVs 30–52 [23], in ZAVs 10–75 [23], in 10-year-old ZAV 86 [19], and in dried KuV 306 [28].		
Phenylalanine (Phe)	Mean content (mg/100 g) of Phe in SAVs 54–70 [23], in ZAVs 19–66 [23], in 10-year-old ZAV 50 [19], and in dried KuV 346 [28].		
Tryptophan (Trp)	Mean content of Trp in dried KuV 17 mg/100 g [28].		
Cysteine (Cys)	Mean content (mg/100 g) of Cys in SAVs 21–30 [23], in ZAVs 10–53 [23], and in dried KuV 226 [28].		
Arginine (Arg)	Mean content (mg/100 g) of Arg in SAVs 37–51 [23], in ZAVs 24–85 [23], in 10-year-old ZAV 16 [19], and in dried KuV 600 [28].		
Aspartic acid (Asp)	Mean content of Asp in dried KuV 828 mg/100 g [28].		
Tyrosine (Tyr)	Mean content (mg/100 g) of Tyr in SAVs 31–51 [23], in ZAVs 12–56 [23], in 10-year-old ZAV 29 [19], and in dried KuV 184 [28].		
Lysine (Lys)	Mean content (mg/100 g) of Lys in SAVs 46–101 [23], in ZAVs 48–72 [23], in 10-year-old ZAV 47 [19], and in dried KuV 223 [28].		
Other amino acids at lower levels: allo-isoleucine, ornithine, taurine, etc.	Total mean amino acid content in SAVs 15.3–28.8 g/L (the highest being that of aged SoV) [23]. Total mean amino acid content in ZAVs 12.2–24.7 g/L (the highest being that of ZHV) [23]. Total mean amino acid content (g/L) in TDMVs 13.1, in FMVs 6.2, in SBrVs 12.0, in ZAVs 8.8, and in SAVs 7.6 [16]. More than 1.8 g/100 g of bioactive branched amino acids (leucine, isoleucine, and valine) in freeze-dried black vinegar powder [15].		
γ-aminobutyric acid (GABA)	GABA was one of the main amino acids in SBrV, along with Ala and Leu [16]. GABA in vinegars fermented from germinated pigmented rice was in the range 1.14–1.73 mg/L (the highest content in germinated Hom Nil rice vinegar) [29].	Inhibitory neurotransmitter in brain. Improvement of cerebral blood flow and cell metabolism. Anti-anxiety and tranquilizing effects. Diouretic. Lipid levels and blood pressure lowering.	ZAV, SAV, TDMV, FMV, SBrV [16,22,29]
**Phenolic acids**			
Vanillic acid (VA)	Mean content (mg/L) of VA in SAVs (SBBs) 18.7–110.8 [17], in ZAV (glutinous rice/bran) 13.3 [17], in 6-year-old ZAV 148.4 [30,31], in 0–7-year-old traditional ZAVs 5.8 [18], in 0–7-year-old industrial ZAVs 7.6 [18], in aged XFV 1.5 [30], in 10-year-old ZAV 15.2 [19], and in 10-year-old ZAV extract 57.0 (prevailing phenolic compound in most cereal vinegars) [30].	Antioxidant propeties—oxidative stress reduction. Antitumor and hepatoprotective effects. Antidiabetic (regulation of glucose metabolism, and amelioration of high glucose-induced insulin resistance).	RV (unpolished, black), SAVs, SoV, BrV, BaV, SBB, KuV, ZAVs (traditional, industrial, aged and non-aged), XFV, ZAV extract (5:15 in ethanol) [9,15,17,18,19,22,28,30,31,32,33,34,35,36]
p-hydroxybenzoic acid (p-HBA)	Mean content (mg/L) of p-HBA in 6-year-old ZAV 27.1 [31], in 0–7-year-old traditional ZAVs 7.5 [18], in 0–7-year-old industrial ZAVs 4.2 [18], in 10-year-old ZAV 24.7 [19], in 10-year-old ZAV extract 30.6 [30], in rice vinegar 5.0 [36], and in dried KuV 1.6 g/100 g [28].		
Gallic acid (GA)	Mean content of GA (mg/L) in SAVs (SBBs) 89.8–252.4 [17], in ZAV (glutinous rice/bran) 555.3 [17], in 10-year-old ZAV 1.0 [19], in 10-year ZAV extract 15.6 [30], in aged XFV 5.9 [35], in rice vinegar 3.4 [36], and in dried KuV 1.3 g/100 g [28].		
Chlorogenic acid (ChA)	Mean content of ChA (mg/L) in SAVs (SBBs) 12.7–20.8 [17], in ZAV (glutinous rice/bran) 7.4 [17], in 0–7-year-old traditional ZAVs 7.0 [18], in 0–7-year-old industrial ZAVs 6.6 [18], in 10-year-old ZAV 6.1 [19], in 10-year ZAV extract 12.0 [30], and in dried KuV 2.6 g/100 g [28].		
Caffeic acid (CA)	Mean content of CA (mg/L) in SAVs (SBBs) 17.5 [17], in 0–7-year-old traditional ZAVs 5.3 [18], in 0–7-year-old industrial ZAVs 5.3 [18], and in 10-year-old ZAV extract 6.6 [30].		
Syringic acid (SyA)	Mean content of SyA (mg/L) in 0–7-year-old traditional ZAVs 12.8 [18], in 0–7-year-old industrial ZAVs 3.1 [18], in 10-year-old ZAV 2.1 [19], in 10-year-old ZAV extract 13.5 [30], and in rice vinegar 1.5 [36].		
p-coumaric acid (p-CoA)	Mean content of p-CoA (mg/L) in SAVs (SBBs) 8.3–9.3 [17], in ZAV (glutinous rice/bran) 2.6 [17], in 0–7-year-old traditional ZAVs 0.7 [18], in aged XFV 0.1 [35], in 10-year-old ZAV 1.7 [19], in 10-year-old ZAV extract 4.4 [30], and in dried KuV 1.8 g/100 g [28].		
Trans-ferulic acid (FA)	Mean content of FA (mg/L) in SAVs (SBBs) 2.5–9.2 [17], in ZAV (glutinous rice/bran) 5.6 [17], in 0–7-year-old traditional ZAVs 6.7 [18], in 0–7-year-old industrial ZAVs 2.8 [18], in 10-year-old ZAV 1.6 [19], in aged XFV 0.2 [35], in 10-year-old ZAV extract 10.2 [30], and in dried KuV 1.5 g/100 g [28].		
Sinapic acid (SiA)	Mean content of SiA (mg/L) in 0–7-year-old traditional ZAVs 3.6 [18], in 0–7-year-old industrial ZAVs 2.8 [18], in 10-year-old ZAV 1.4 [19], in 10-year-old ZAV extract 4.8 [30], and in dried KuV 1.6 g/100 g [28].		
Protocatechuic acid (PA)	Mean content of PA 4.1 mg/L in rice vinegar [36].		
Salicylic acid (SaA)	Mean content of SaA 7.2 mg/L in SAVs (SBBs) [17].		
Other acids: o-coumaric, dihydroferulic, dihydrosinapic, gentisic, 3-hydroxyphenylacetic, isoferulic, 3-methylsalicylic, 3-phenylacetic, phloretic, phloroglucinic, 2,4,5-trihydroxybenzoic, etc.	Total phenolic acid content in 5-year-old SAV up to 980 ± 11 μg/g as gallic acid [32].		
**Flavonoids and other phenolics**			
Catechin	Mean content (mg/L) of catechin (as (±)-catechin hydrate) in SAVs (SBB) 112.7–139.5 and in ZAV (glutinous rice/bran) 34.0 [17], in 6-year-old ZAV 57.5 [31], in 10-year-old ZAV 36.8 [19], in 0–7-year-old traditional ZAVs 41.4 [18], in 0–7-year-old industrial ZAVs 64.2 [18], in 10-year-old ZAV extract 133.2 [30], in rice vinegar 3.6 [36], and in dried KuV 2.3 g/100 g [28].	Antioxidant properties—oxidative stress reduction. Antitumor properties. Hepatoprotective effects. Anti-inflammatory properties. Improvement of gut microbiota-related disorders. Inhibition of amylolytic enzyme activities and dietary carbohydrate absorption. Antioxidant activities in the food matrix (prevention of lipid oxidation, color, taste, and texture preservation).	SAV cereal vinegars (SoV, OaV, TBV, SBB, etc.), ZAV (ZHV, LRV, traditional, industrial, aged and non-aged), BRV, KuV [17,18,19,23,24,28,30,31,33,34,37,38].
Rutin	Mean content (mg/L) of rutin in 10-year-old ZAV 1.8 [19], in 0–7-year-old traditional ZAVs 13.1 [18], and in 10-year-old ZAV extract 22.3 [30].		
L-epicatechin	Mean content of epicatechin 13.3 mg/L in ZAV (glutinous rice/bran) [17].		
(-)-epicatechin gallate	Mean content of epicatechin gallate 37.9 mg/L in SAVs (SBB) [17].		
Anthocyanins	Mean anthocyanin content in BRV 10.9 mg/L [37].		
Other phenolics: o-cresol, syringaldehyde, syringol, sinapaldehyde, vanillin, etc.			
Total phenolic content (TPC), flavonoid content (TFC), and antioxidant capacity (AC)	TPC, TFC, and AC in cereal vinegars generally increase with brewing and aging time [24]. Mean TPC (mg GAE/mL) were found to be in SAVs 2.8 (OaV), 39.7 (SoV) and 45.2 (TBV) [23], in SAVs (SBB) 2.17 and in ZAV (glutinous rice/bran) 1.46 [17], in 1–10 years aged SAVs 1.0–5,8 [24], in 0–7 years aged traditional ZAV 4.3 [18], and in ZAVs 1.6 (ZHV) and 0.38 (LRB) [23]. Mean TFC (mg/L) in SAVs (SBB) was 21.0 [17], in 0–7 years aged traditional ZAV 320 [18], and in 1–10 years aged SAVs 330–4500 mg/kg [24].		
**Non-enzymatic browning reaction products**			
Melanoidins	A mean browning index (A420 nm) of 0.26–0.83 for high MW melanoidins in 0–6-year-old ZAV [31].	Antioxidant properties. Antiobesity properties. Hepatoprotective effects (melanoidins). Color, texture and flavor in vinegars. Possible negative health effects at high levels (mainly HMF and 2-F).	KuV, ZAV, SAV, FMV, TDMV [16,18,31,39,40,41]
5-hydroxymethylfurfural (HMF)	HMF content (mg/L) in ZAVs 61.8–455.1, in SAVs 35.8–205.7, in FMVs 26.8–59.5, and mean content in TDMVs 92.4 [16].		
2-furfural (2-F)	2-F content (mg/L) in ZAVs 110.3–272.2, in SAVs 6.3–290.6, in FMVs 4.4–5.7, and in TDMVs (av.) 114.3 [16].		
**Alkaloids**			
Saponin/total alkaloids	Mean levels in cereal SAVs (mg/mL): saponin 0.59 (in SoV), 0.66 (in OaV), 0.60 (in TBV), and total alkaloids 0.82 (in SoV), 1.21 (in OaV), 0.65 (in TBV) [23]. Mean levels in ZAVs (mg/mL): saponin 0.29 (in ZHV), 0.18 (in LRV), and total alkaloids 0.98 (in ZHV), 0.09 (in LRV) [23].	Antioxidant properties. Anti-inflammatory properties. Antimicrobial and antimalarial properties. Hypouricemic effects. Anticarcinogenic properties. Neuromodulatory effects (inhibition of the antidepressant monoamine oxidase and benzodiazepine receptor). Negative health effects include neurotoxicity and genotoxicity.	RV, SAV cereal vinegars (SoV, OaV, TBV), ZAV (ZHV, LRV), Taiwan SoV [22,23,27,35,42,43,44,45,46].
β-carbolines [1,2,3,4-tetrahydro-β-carboline-3-carboxylic acid (THCA) and 1-methyl-1,2,3,4-tetrahydro-β-carboline-3-carboxylic acid (MTCA)]	Mean β-carbolines content (μg/L) THCA 50, MTCA-SS 90, and MTCA-RS 1380 in SoV (Taiwan) [27].		
2,3,5,6-tetramethylpyrazine (ligustrazine)	Above 30 mg/L in SAV according to national standards [42]. Mean content (mg/L) 6.4 in non-aged XFV, 3.3 in aged XFV [35], and 697 in 6-year-old ZAV [43].	Inhibition of reactive species. Increase in antioxidant enzymes. Inhibition of platelet aggregation. Blood vessel vasodilation. Reduction in blood lipids. Hepatoprotective effects. Aroma contributor in vinegar (peanut, nutty).	TBV, SAV, ZAV, BrV, FV [22,35,42,43,44,45,46].
**Butenolides (4-C heterocyclic ring lactones)**			
Fraglide-1 (5-Hydroxy-4-phenyl-butenolide)	Content (μg/L) in brown rice vinegar (KuV) 19.99 ± 3.11, in 6 months aged Kozu 164–1336, in 8 years aged Kozu 239–1987, and in Chinese sticky rice husk 122.2 ± 0.7 [47].	Anticancer properties. Antimicrobial and antiviral properties. Anti-inflammatory properties. Regulation of fatty acid storage and glucose metabolism (agonist for PPARγ). Antiobesity effects.	ZAV (Kozu), KuV, Chinese sticky rice husk [47].
**γ-Oryzanol**			
Mixture of triterpene alcohol and phytosterol ferulates	Not reported in cereal vinegars. Total content in Chinese rice varieties was 10.9–33.5 mg/100 g [48]. Cycloartenol ferulate, 24-methylenecycloartanyl, campesterol ferulate and β-sitosterol ferulate were the most abundant consituents [48]. Mean content (mg/100 g) of γ-oryzanol in brown rice from Wuchang was 21.5 ± 5.0, and 16.6 ± 6.3 in brown rice from Xiangshui [48].	Antioxidant, anticarginogenic, anti-inflammatory, antihyperlipidemic, and neuroprotective properties. Inhibition of lipid peroxidation in food.	Rice [48].

Rice vinegars (RV); Shanxi aged vinegar (SAV); Zhenjiang aromatic vinegar (ZAV); Sorghum vinegar (SoV); Oat vinegar (OaV); Sorghum-barley-Bran vinegar (SBB); Tartary buckwheat vinegar (TBV); Zhenjiang Hengshun vinegar (ZHV); Liubiju rice vinegar (LRV); Fujian Monascus vinegar (FMV), Sichuan bran vinegar (SBrV); Tianjin duliu mature vinegar (TDMV); Kurosu vinegar (KuV); Bran vinegar (BrV); Barley vinegar (BaV); Wheat bran vinegar (WBrV); Broken Riceberry rice vinegar (BRV); Xiangxi flavor vinegar (XFV); total flavonoid content (TFC); total phenolic content (TPC); gallic acid equivalent (GAE); rutin equivalent (RE); peroxisome proliferator-activated receptor γ (PPARγ).

## Data Availability

Data sharing not applicable.

## References

[1] Lim S.J., Ho C.W., Lazim A.M., Fazry S., Bekatorou A. (2019). History and current issues of vinegar. Advances in Vinegar Production.

[2] Lazim A.M., Lim S.J., Ho C.W., Fazry S., Bekatorou A. (2019). Types of vinegars. Advances in Vinegar Production.

[3] Kandylis P., Bekatorou A. (2019). Innovative vinegar products. Advances in Vinegar Production.

[4] Zhang Q., Fu C., Zhao C., Yang S., Zheng Y., Xia M., Yan Y., Lang F., Wang M. (2020). Monitoring microbial succession and metabolic activity during manual and mechanical solid-state fermentation of Chinese cereal vinegar. LWT.

[5] Li W., Fan G., Fu Z., Wang W., Xu Y., Teng C., Zhang C., Yang R., Sun B., Li X. (2020). Effects of fortification of Daqu with various yeasts on microbial community structure and flavor metabolism. Food Res. Int..

[6] Nie Z., Zheng Y., Xie S., Zhang X., Song J., Xia M., Wang M. (2017). Unraveling the correlation between microbiota succession and metabolite changes in traditional Shanxi aged vinegar. Sci. Rep..

[7] Wu L.H., Lu Z.M., Zhang X.J., Wang Z.M., Yu Y.J., Shi J.S., Xu Z.H. (2017). Metagenomics reveals flavour metabolic network of cereal vinegar microbiota. Food Microbiol..

[8] Ling J.W.A., Mun S.L., Fazry S., Lazim A.M., Lim S.J., Bekatorou A. (2019). Health benefits of vinegars. Advances in Vinegar Production.

[9] Budak N.H., Aykin E., Seydim A.C., Greene A.K., Guzel-Seydim Z.B. (2014). Functional properties of vinegar. J. Food Sci..

[10] Martillanes S., Rocha-Pimienta J., Gil M.V., Ayuso-Yuste M.C., Delgado-Adámez J. (2020). Antioxidant and antimicrobial evaluation of rice bran (*Oryza sativa* L.) extracts in a mayonnaise-type emulsion. Food Chem..

[11] Lemus C., Angelis A., Halabalaki M., Skaltsounis A.L., Watson R.R., Preedy V.R., Zibadi S. (2014). γ-Oryzanol. An attractive bioactive component from rice bran. Wheat and Rice in Disease Prevention and Health.

[12] Castanho A., Lageiro M., Calhelha R.C., Ferreira I.C.F.R., Sokovic M., Cunha L.M., Brites C. (2019). Exploiting the bioactive properties of γ-oryzanol from bran of different exotic rice varieties. Food Funct..

[13] Hajilou H., Farahpour M.R., Hamishehkar H. (2020). Polycaprolactone nanofiber coated with chitosan and gamma oryzanol functionalized as a novel wound dressing for healing infected wounds. Int. J. Biol. Macromol..

[14] Lomthong T., Saithong P. (2019). Feasibility of Leum Pua glutinous rice substrate for sugar syrup and vinegar production by raw starch degrading enzyme hydrolysis. Int. Food Res. J..

[15] Liu M.E., Chou C.H., Li L., Wu Y.H.S., Lin Y.L., Tu D.G., Chen Y.C. (2020). Modulation effects of black-vinegar-based supplement against a high-fat dietary habit: Antiobesity/hypolipidemic, antioxidative, and energy-metabolism effects. J. Sci. Food Agric..

[16] Gong M., Zhou Z., Yu Y., Liu S., Zhu S., Jian D., Cui P., Zhong F., Mao J. (2020). Investigation of the 5-hydroxymethylfurfural and furfural content of Chinese traditional fermented vinegars from different regions and its correlation with the saccharide and amino acid content. LWT.

[17] Ren M., Wang X., Tian C., Li X., Zhang B., Song X., Zhang J. (2017). Characterization of organic acids and phenolic compounds of cereal vinegars and fruit vinegars in China. J. Food Process. Pres..

[18] Zhao C., Xia T., Du P., Duan W., Zhang B., Zhang J., Zhu S., Zheng Y., Wang M., Yu Y. (2018). Chemical composition and antioxidant characteristic of traditional and industrial Zhenjiang aromatic vinegars during the aging process. Molecules.

[19] Zhang B., Xia T., Duan W., Zhang Z., Li Y., Fang B., Xia M., Wang M. (2019). Effects of organic acids, amino acids and phenolic compounds on antioxidant characteristic of Zhenjiang aromatic vinegar. Molecules.

[20] Zhao G., Kuang G., Li J., Hadiatullah H., Chen Z., Wang X., Yao Y., Pan Z.H., Wang Y. (2020). Characterization of aldehydes and hydroxy acids as the main contribution to the traditional Chinese rose vinegar by flavor and taste analyses. Food Res. Int..

[21] Zheng Y., Cheng C., Liu J., Liu X., Xie X., Xu Y., Song J., Wang M. (2020). Analysis of main characteristic flavor components in Chinese traditional solid-state fermented vinegars. J. Chin. Inst. Food Sci. Tech..

[22] Xia T., Zhang B., Duan W., Zhang J., Wang M. (2020). Nutrients and bioactive components from vinegar: A fermented and functional food. J. Funct. Foods.

[23] Fan J., Zhang Y., Zhou L., Li Z., Zhang B., Saito M., Wang X. (2011). Nutritional composition and α-glucosidase inhibitory activity of five Chinese vinegars. Jpn. Agr. Res. Q. JARQ.

[24] Xie X., Zheng Y., Liu X., Cheng C., Zhang X., Xia T., Yu S., Wang M. (2017). Antioxidant activity of Chinese Shanxi aged vinegar and its correlation with polyphenols and flavonoids during the brewing process. J. Food Sci..

[25] Kim H., Lee H., Shin K.S. (2016). Intestinal immunostimulatory activity of neutral polysaccharide isolated from traditionally fermented Korean brown rice vinegar. Biosci. Biotechnol. Biochem..

[26] Li J., Yu G., Fan J. (2014). Alditols and monosaccharides from sorghum vinegar can attenuate platelet aggregation by inhibiting cyclooxygenase-1 and thromboxane-A2 synthase. J. Ethnopharmacol..

[27] Chiu H.F., Cheng Y., Lu Y.Y., Han Y.C., Shen Y.C., Venkatakrishnan K., Wang C.K. (2017). Anti-mutagenicity, hypouricemic and antioxidant activities of alkaloids from vinegar and mei vinegar. J. Food Biochem..

[28] Chou C.H., Liu C.W., Yang D.J., Wu Y.H.S., Chen Y.C. (2015). Amino acid, mineral, and polyphenolic profiles of black vinegar, and its lipid lowering and antioxidant effects in vivo. Food Chem..

[29] Phuapaiboon P. (2017). Gamma-aminobutyric acid, total anthocyanin content and antioxidant activity of vinegar brewed from germinated pigmented rice. Pakistan J. Nutr..

[30] Xia T., Duan W., Zhang Z., Fang B., Zhang B., Xu B., de la Cruz C.B.V., El-Seedi H., Simal-Gandara J., Wang S. (2021). Polyphenol-rich extract of Zhenjiang aromatic vinegar ameliorates high glucose-induced insulin resistance by regulating JNK-IRS-1 and PI3K/Akt signaling pathways. Food Chem..

[31] Duan W., Xia T., Zhang B., Li S., Zhang C., Zhao C., Song J., Wang M. (2019). Changes of physicochemical, bioactive compounds and antioxidant capacity during the brewing process of Zhenjiang aromatic vinegar. Molecules.

[32] Chen H., Zhou Y., Shao Y., Chen F. (2016). Free phenolic acids in Shanxi aged vinegar: Changes during aging and synergistic antioxidant activities. Int. J. Food Prop..

[33] Xia T., Zhang B., Duan W., Li Y., Zhang J., Song J., Zheng Y., Wang M. (2019). Hepatoprotective efficacy of Shanxi aged vinegar extract against oxidative damage in vitro and in vivo. J. Funct. Foods.

[34] Ho C.W., Lazim A.M., Fazry S., Zaki U.K.H.H., Lim S.J. (2017). Varieties, production, composition and health benefits of vinegars: A review. Food Chem..

[35] Huang R.T., Huang Q., Wu G.L., Chen C.G., Li Z.J. (2017). Evaluation of the antioxidant property and effects in *Caenorhabditis elegans* of Xiangxi flavor vinegar, a Hunan local traditional vinegar. J. Zhejiang Univ. Sci. B.

[36] Bakir S., Devecioglu D., Kayacan S., Toydemir G., Karbancioglu-Guler F., Capanoglu E. (2017). Investigating the antioxidant and antimicrobial activities of different vinegars. Eur. Food Res. Technol..

[37] Sangngern N., Puangnark T., Nguansangiam W., Saithong P., Kitpreechavanich V., Lomthong T. (2020). Production and development of vinegar fermentation from broken Riceberry rice using raw starch-degrading enzyme hydrolysis. 3 Biotech..

[38] Du P., Zhou J., Zhang L., Zhang J., Li N., Zhao C., Tu L., Zheng Y., Xia T., Luo J. (2020). GC × GC-MS analysis and hypolipidemic effects of polyphenol extracts from Shanxi-aged vinegar in rats under a high fat diet. Food Funct..

[39] Xia T., Zhang J., Yao J., Zhang B., Duan W., Xia M., Song J., Zheng Y., Wang M. (2018). Shanxi aged vinegar prevents alcoholic liver injury by inhibiting CYP2E1 and NADPH oxidase activities. J. Funct. Foods.

[40] Suzuki E., Otake S., Hamadate N., Hasumi K. (2020). Kurozu melanoidin, a novel oligoglucan-melanoidin complex from Japanese black vinegar, suppresses adipogenesis in vitro. J. Funct. Foods.

[41] Chen H., Wang S., Fu H., Chen F., Zhang L., Lan W., Yang J., Yang X., She Y. (2020). A colorimetric sensor array for recognition of 32 Chinese traditional cereal vinegars based on “turn-off/on” fluorescence of acid-sensitive quantum dots. Spectrochim. Acta-Part A Mol. Biomol. Spectrosc..

[42] Chai L.J., Qiu T., Lu Z.M., Deng Y.J., Zhang X.J., Shi J.S., Xu Z.H. (2020). Modulating microbiota metabolism via bioaugmentation with *Lactobacillus casei* and *Acetobacter pasteurianus* to enhance acetoin accumulation during cereal vinegar fermentation. Food Res. Int..

[43] Xu W., Xu Q., Chen J., Lu Z., Xia R., Li G., Xu Z., Ma Y. (2011). Ligustrazine formation in Zhenjiang aromatic vinegar: Changes during fermentation and storing process. J. Sci. Food Agr..

[44] Chen J., Tian J., Ge H., Liu R., Xiao J. (2017). Effects of tetramethylpyrazine from Chinese black vinegar on antioxidant and hypolipidemia activities in HepG2 cells. Food Chem. Toxicol..

[45] Zhang L., Huang J., Zhou R., Wu C. (2017). Evaluating the feasibility of fermentation starter inoculated with Bacillus amyloliquefaciens for improving acetoin and tetramethylpyrazine in Baoning bran vinegar. Int. J. Food Microbiol..

[46] Zhou Z., Jian D., Gong M., Zhu S., Li G., Zhang S., Zhong F., Mao J. (2020). Characterization of the key aroma compounds in aged Zhenjiang aromatic vinegar by gas chromatography-olfactometry-mass spectrometry, quantitative measurements, aroma recombination and omission experiments. Food Res. Int..

[47] Yatmaz A.H., Kinoshita T., Miyazato A., Takagi M., Tsujino Y., Beppu F., Gotoh N. (2017). Quantification of Fraglide-1, a new functional ingredient, in vinegars. J. Oleo Sci..

[48] Zhang C., Li D., Zhang C., Li H., Yin K., Zhang X., Zhang D. (2020). γ-Oryzanol content and ferulic acid ester composition in brown rice from Heilongjiang Province. Food Sci..

[49] Pisoschi A.M., Pop A., Iordache F., Stanca L., Predoi G., Serban A.I. (2021). Oxidative stress mitigation by antioxidants—An overview on their chemistry and influences on health status. Eur. J. Med. Chem..

[50] Maes M., Galecki P., Chang Y.S., Berk M. (2011). A review on the oxidative and nitrosative stress (OandNS) pathways in major depression and their possible contribution to the (neuro) degenerative processes in that illness. Prog. Neuro Psychoph..

[51] Liu J., Gan J., Nirasawa S., Zhou Y., Xu J., Zhu S., Cheng Y. (2017). Cellular uptake and trans-enterocyte transport of phenolics bound to vinegar melanoidins. J. Funct. Foods.

[52] Shimoji Y., Tamura Y., Nakamura Y., Nanda K., Nishidai S., Nishikawa Y., Ohigashi H. (2002). Isolation and identification of DPPH radical scavenging compounds in Kurosu (Japanese unpolished rice vinegar). J. Agr. Food Chem..

[53] Shimoji Y., Kohno H., Nanda K., Nishikawa Y., Ohigashi H., Uenakai K., Tanaka T. (2004). Extract of Kurosu, a vinegar from unpolished rice, inhibits azoxymethane-induced colon carcinogenesis in male F344 rats. Nutr. Cancer.

[54] Shibayama Y., Kanouchi H., Fujii A., Nagano M. (2020). A review of Kurozu, amber rice vinegar made in pottery jars. Funct. Foods Health Dis..

[55] Nanda K., Miyoshi N., Nakamura Y., Shimoji Y., Tamura Y., Nishikawa Y., Uenakai K., Kohno H., Tanaka T. (2004). Extract of vinegar “Kurosu” from unpolished rice inhibits the proliferation of human cancer cells. J. Exp. Clin. Cancer Res..

[56] Nagashima M., Saito K. (2010). Antioxidant activity of the new black vinegar “iZUMI”. J. Nutr. Health Aging.

[57] Xia T., Yao J.H., Zhang J., Duan W.H., Zhang B., Xie X.L., Xia M.L., Song J., Zheng Y., Wang M. (2018). Evaluation of nutritional compositions, bioactive compounds, and antioxidant activities of Shanxi aged vinegars during the aging process. J. Food Sci..

[58] Gao Y., Jo Y., Chung N., Gu S.-Y., Jeong Y.-J., Kwon J.-H. (2017). Physicochemical qualities and flavor patterns of traditional Chinese vinegars manufactured by different fermentation methods and aging periods. Prev. Nutr. Food Sci..

[59] Choi H., Gwak G., Choi D., Park J., Cheong H. (2015). Antimicrobial efficacy of fermented dark vinegar from unpolished rice. Microbiol. Biotechnol. Lett..

[60] Yu X., Yang M., Dong J., Shen R. (2018). Comparative analysis of the antioxidant capacities and phenolic compounds of oat and buckwheat vinegars during production processes. J. Food Sci..

[61] Pazuch C.M., Kalschne D.L., Siepmann F.B., Marx I.M.G., de Oliveira T.C.G., Spinosa W.A., Canan C., Colla E. (2020). Optimization and characterization of vinegar produced from rice bran. Food Sci. Technol..

[62] Li Y., He Y., Hu H., Li H., Hu J., Cheng Z. (2018). Study of antioxidant, thrombolytic and antithrombus activities of Tartary buckwheat vinegar. J. Chin. Inst. Food Sci. Technol..

[63] Mudura E., Coldea T.E., Socaciu C., Ranga F., Pop C.R., Rotar A.M., Pasqualone A. (2018). Brown beer vinegar: A potentially functional product based on its phenolic profile and antioxidant activity. J. Serb. Chem. Soc..

[64] Ho C.W., Lazim A.M., Fazry S., Hussain Zaki U.M.K.H., Lim S.J. (2017). Effects of fermentation time and pH on soursop (Annona muricata) vinegar production towards its chemical compositions. Sains Malays..

[65] Ali Z., Wang Z., Amir R.M., Younas S., Wali A., Adowa N., Ayim I. (2016). Potential uses of vinegar as a medicine and related in vivo mechanisms. Int. J. Vitam. Nutr. Res..

[66] Bouarab-Chibane L., Forquet V., Lantéri P., Clément Y., Léonard-Akkari L., Oulahal N., Degraeve P., Bordes C. (2019). Antibacterial properties of polyphenols: Characterization and QSAR (Quantitative Structure–Activity Relationship) models. Front. Microbiol..

[67] Peh E., Kittler S., Reich F., Kehrenberg C. (2020). Antimicrobial activity of organic acids against Campylobacter spp. and development of combinations—A synergistic effect?. PLoS ONE.

[68] Kadiroğlu P. (2018). FTIR spectroscopy for prediction of quality parameters and antimicrobial activity of commercial vinegars with chemometrics. J. Sci. Food Agric..

[69] Carpenter C.E., Broadbent J.R. (2009). External concentration of organic acid anions and pH: Key independent variables for studying how organic acids inhibit growth of bacteria in mildly acidic foods. J. Food Sci..

[70] Mani-López E., García H.S., López-Malo A. (2012). Organic acids as antimicrobials to control *Salmonella* in meat and poultry products. Food Res. Int..

[71] Pinto L., Caputo L., Quintieri L., de Candia S., Baruzzi F. (2017). Efficacy of gaseous ozone to counteract postharvest table grape sour rot. Food Microbiol..

[72] Pinto L., Malfeito-Ferreira M., Quintieri L., Silva A.C., Baruzzi F. (2019). Growth and metabolite production of a grape sour rot yeast-bacterium consortium on different carbon sources. J. Food Microbiol..

[73] Chai L.J., Shen M.N., Sun J., Deng Y.J., Lu Z.M., Zhang X.J., Shi J.S., Xu Z.H. (2020). Deciphering the D-/l-lactate-producing microbiota and manipulating their accumulation during solid-state fermentation of cereal vinegar. Food Microbiol..

[74] Wang Q., Buchanan R.L., Tikekar R.V. (2019). Evaluation of adaptive response in *E. coli* O157:H7 to UV light and gallic acid based antimicrobial treatments. Food Control.

[75] Sinsinwar S., Vadivel V. (2020). Catechin isolated from cashew nut shell exhibits antibacterial activity against clinical isolates of MRSA through ROS-mediated oxidative stress. Appl. Microbiol. Biot..

[76] Lekbach Y., Dong Y., Li Z., Xu D., El Abed S., Yi Y., Li L., Ibnsouda Koraichi S., Sun T., Wang F. (2019). Catechin hydrate as an eco-friendly biocorrosion inhibitor for 304 L stainless steel with dual-action antibacterial properties against *Pseudomonas aeruginosa* biofilm. Corros. Sci..

[77] Bernal-Mercado A.T., Vazquez-Armenta F.J., Tapia-Rodriguez M.R., Islas-Osuna M.A., Mata-Haro V., Gonzalez-Aguilar G.A., Lopez-Zavala A.A., Ayala-Zavala J.F. (2018). Comparison of single and combined use of catechin, protocatechuic, and vanillic acids as antioxidant and antibacterial agents against uropathogenic *Escherichia coli* at planktonic and biofilm levels. Molecules.

[78] Qian W., Fu Y., Liu M., Wang T., Zhang J., Yang M., Sun Z., Li X., Li Y. (2019). In vitro antibacterial activity and mechanism of vanillic acid against carbapenem-resistant *Enterobacter cloacae*. Antibiotics.

[79] Qian W., Yang M., Wang T., Sun Z., Liu M., Zhang J., Zeng Q., Cai C., Li Y. (2020). Antibacterial mechanism of vanillic acid on physiological, morphological, and biofilm properties of carbapenem-resistant *Enterobacter hormaechei*. J. Food Prot..

[80] Heidtmann-Bemvenuti R., Tralamazza S.M., Jorge Ferreira C.F., Corrêa B., Badiale-Furlong E. (2016). Effect of natural compounds on *Fusarium graminearum* complex. J. Sci. Food Agric..

[81] Chang J.M., Fang T.J. (2007). Survival of *Escherichia coli* O157:H7 and *Salmonella enterica* serovars Typhimurium in iceberg lettuce and the antimicrobial effect of rice vinegar against *E. coli* O157:H7. Food Microbiol..

[82] Maqbul M.S., Alshabi A.M., Khan A.A., Iqubal S.S., Mohammed T., Shaikh I.A., Dawoud A., Muddapur U.M., Hussain M.S., Singh S.K. (2020). Comparison of e-test values for standard antibiotics and conventional antimicrobial assay values for ethanoic acids against nosocomial multidrug-resistant *Pseudomonas aeruginosa*. J. Pure Appl. Microbiol..

[83] Krusong W., Sriphochanart W., Suwapanich R., Mekkerdchoo O., Sriprom P., Wipatanawin A., Massa S. (2020). Healthy dried baby corn silk vinegar production and determination of its main organic volatiles containing antimicrobial activity. LWT.

[84] Liu Y., Hannig M. (2020). Vinegar inhibits the formation of oral biofilm in situ. BMC Oral Health.

[85] Ben-Avraham S., Harman-Boehm I., Schwarzfuchs D., Shai I. (2009). Dietary strategies for patients with type 2 diabetes in the era of multi-approaches: Review and results from the Dietary Intervention Randomized Controlled Trial (DIRECT). Diabetes Res. Clin. Pract..

[86] Johnston C.S., Buller A.J. (2005). Vinegar and peanut products as complementary foods to reduce postprandial glycemia. J. Am. Diet. Assoc..

[87] Johnston C.S., Gaas C.A. (2006). Vinegar: Medicinal uses and antiglycemic effect. Med. Gen. Med..

[88] Santos H.O., de Moraes W.M., da Silva G.A., Prestes J., Schoenfeld B.J. (2019). Vinegar (acetic acid) intake on glucose metabolism: A narrative review. Clin. Nutr. ESPEN.

[89] Lim J., Henry C.J., Haldar S. (2016). Vinegar as a functional ingredient to improve postprandial glycemic control-human intervention findings and molecular mechanisms. Mol. Nutr. Food Res..

[90] Fushimi T., Suruga K., Oshima Y., Fukiharu M., Tsukamoto Y., Goda T. (2006). Dietary acetic acid reduces serum cholesterol and triacylglycerols in rats fed a cholesterol-rich diet. Br. J. Nutr..

[91] Fushimi T., Sato Y. (2005). Effect of acetic acid feeding on the circadian changes in glycogen and metabolites of glucose and lipid in liver and skeletal muscle of rats. Br. J. Nutr..

[92] Mitrou P., Petsiou E., Papakonstantinou E., Maratou E., Lambadiari V., Dimitriadis P., Spanoudi F., Raptis S.A., Dimitriadis G. (2015). Vinegar consumption increases insulin-stimulated glucose uptake by the forearm muscle in humans with type 2 diabetes. J. Diabetes Res..

[93] Mitrou P., Petsiou E., Papakonstantinou E., Maratou E., Lambadiari V., Dimitriadis P., Spanoudi F., Raptis S.A., Dimitriadis G. (2015). The role of acetic acid on glucose uptake and blood flow rates in the skeletal muscle in humans with impaired glucose tolerance. Eur. J. Clin. Nutr..

[94] Pan J.H., Kim J.H., Kim H.M., Lee E.S., Shin D.H., Kim S., Shin M., Kim S.H., Lee J.H., Kim Y.J. (2015). Acetic acid enhances endurance capacity of exercise-trained mice by increasing skeletal muscle oxidative properties. Biosci. Biotechnol. Biochem..

[95] Ogawa N., Satsu H., Watanabe H., Fukaya M., Tsukamoto Y., Miyamoto Y., Shimizu M. (2000). Acetic acid suppresses the increase in disaccharidase activity that occurs during culture of caco-2 cells. J. Nutr..

[96] Liljeberg H., Björck I. (1998). Delayed gastric emptying rate may explain improved glycaemia in healthy subjects to a starchy meal with added vinegar. Eur. J. Clin. Nutr..

[97] White A.M., Johnston C.S. (2007). Vinegar ingestion at bedtime moderates waking glucose concentrations in adults with well-controlled type 2 diabetes. Diab. Care.

[98] Papoutsis K., Zhang J., Bowyer M.C., Brunton N., Gibney E.R., Lyng J. (2021). Fruit, vegetables, and mushrooms for the preparation of extracts with α-amylase and α-glucosidase inhibition properties: A review. Food Chem..

[99] Hua F., Zhou P., Wu H.Y., Chu G.X., Xie Z.W., Bao G.H. (2018). Inhibition of α-glucosidase and α-amylase by flavonoid glycosides from Lu’an GuaPian tea: Molecular docking and interaction mechanism. Food Funct..

[100] Zheng Y., Tian J., Yang W., Chen S., Liu D., Fang H., Zhang H., Ye X. (2020). Inhibition mechanism of ferulic acid against α-amylase and α-glucosidase. Food Chem..

[101] Gu X., Zhao H.L., Sui Y., Guan J., Chan J.C., Tong P.C. (2012). White rice vinegar improves pancreatic beta-cell function and fatty liver in streptozotocin-induced diabetic rats. Acta Diabetol..

[102] Ma T., Shan F., Changxi J.I.A. (2010). Effects of Tartary buckwheat vinegar on blood glucose of diabetic mice. J. Chin. Cereals Oils Assoc..

[103] Cheng L.J., Jiang Y., Wu V.X., Wang W. (2020). A systematic review and meta-analysis: Vinegar consumption on glycaemic control in adults with type 2 diabetes mellitus. J. Adv. Nurs..

[104] Petsiou E.I., Mitrou P.I., Raptis S.A., Dimitriadis G.D. (2014). Effect and mechanisms of action of vinegar on glucose metabolism, lipid profile, and body weight. Nutr. Rev..

[105] Beheshti Z., Chan Y.H., Nia H.S., Hajihosseini F., Nazari R., Shaabani M. (2012). Influence of apple cider vinegar on blood lipids. Life Sci. J..

[106] Yamashita H., Fujisawa K., Ito E., Idei S., Kawaguchi N., Kimoto M., Hiemori M., Tsuji H. (2007). Improvement of obesity and glucose tolerance by acetate in Type 2 diabetic Otsuka Long-Evans Tokushima Fatty (OLETF) rats. Biosci. Biotechnol. Biochem..

[107] Launholt T.L., Kristiansen C.B., Hjorth P. (2020). Safety and side effects of apple vinegar intake and its effect on metabolic parameters and body weight: A systematic review. Eur. J. Nutr..

[108] Östman E., Granfeldt Y., Persson L., Björck I. (2005). Vinegar supplementation lowers glucose and insulin responses and increases satiety after a bread meal in healthy subjects. Eur. J. Clin. Nutr..

[109] Chen J.C., Zheng B.D., Zhao Y.Y., Xu J., Wu J.J., Luo C., Pang J., He G.Q. (2012). Hypolipidemic effects on lipid metabolism and lipase inhibition by black vinegar powder. J. Biobased Mater. Bioenergy.

[110] Chen H., Chen T., Giudici P., Chen F. (2016). Vinegar functions on health: Constituents, sources, and formation mechanisms. Compr. Rev. Food Sci. Food Saf..

[111] Samad A., Azlan A., Ismail A. (2016). Therapeutic effects of vinegar: A review. Curr. Opin. Food Sci..

[112] Hamadate N., Nakamura K.I., Hirai M., Yamamoto T., Yamaguchi H., Iizuka M., Yamamoto E., Iwama Y., Yazawa K. (2013). Effect of a dietary supplement containing Kurozu (a Japanese traditional health drink) concentrate on several obesity-related parameters in obese Japanese adults: A randomized, double-blind, placebo-controlled trial. Funct. Foods Health Dis..

[113] Hamadate N., Seto K., Yazawa K. (2014). Effects of a Dietary Supplement Containing Kurozu Concentrate on Body Fat and Energy Metabolism. Jpn. J. Complem. Altern. Med..

[114] Nakasone Y., Miura H. (2017). Effect of a traditional Japanese health food made from Kurozu (unrefined black rice vinegar) and garlic combination on serum cholesterol in subjects with prehypercholesterolemia or mild-to-moderate hypercholesterolemia-A randomized, double-blind, placebo-controlled, intervention study. Jpn. Pharmacol. Ther..

[115] Abe S., Hasegawa M., Tsuruoka J., Matsumoto Y., Koyanagi S. (2019). Effect of dietary supplement containing Kurozu concentrate on visceral fat accumulation. Jpn. J. Complem. Altern. Med..

[116] Li B., Li Z., Wei Y., Zhang X., Wu R., Fan Y., Bu L. (2009). Study on the effects of brans and *Aspergillus niger* about corn vinegar on reducing obesity and blood lipids in rat. J. Northwest A F Univ. Nat. Sci. Ed..

[117] Tong L.T., Katakura Y., Kawamura S., Baba S., Tanaka Y., Udono M., Kondo Y., Nakamura K., Imaizumi K., Sato M. (2010). Effects of Kurozu concentrated liquid on adipocyte size in rats. Lipids Health Dis..

[118] Liu L., Yang X. (2015). Hypolipidemic and antioxidant effects of freeze-dried powder of Shanxi mature vinegar in hyperlipidaemic mice. Food Sci..

[119] Shibayama Y., Nagano M., Hashiguchi K., Fujii A., Iseki K. (2019). Supplementation of concentrated Kurozu, a Japanese black vinegar, reduces the onset of hepatic steatosis in mice fed with a high-fat diet. Funct. Foods Health Dis..

[120] Zhao Y., He Z., Hao W., Zhu H., Liang N., Liu J., Zhang C., Ma K.Y., He W.S., Yang Y. (2020). Vinegars but not acetic acid are effective in reducing plasma cholesterol in hamsters fed a high-cholesterol diet. Food Funct..

[121] Ruppin H., Bar-Meir S., Soergel K.H., Wood C.M., Schmitt M.G. (1980). Absorption of short-chain fatty acids by the colon. Gastroenterology.

[122] Martin-Gallausiaux C., Marinelli L., Blottière H.M., Larraufie P., Lapaque N. (2020). SCFA: Mechanisms and functional importance in the gut. Proc. Nutr. Soc..

[123] Tasdemir S.S., Sanlier N. (2020). An insight into the anticancer effects of fermented foods: A review. J. Funct. Foods.

[124] Moloney J.N., Cotter T.G. (2018). ROS signalling in the biology of cancer. Semin. Cell Dev. Biol..

[125] Chen F., Gullo M. Acetic acid bacteria. Proceedings of the 4th International Conference on Acetic Acid Bacteria—Vinegar and Other Products (AAB 2015).

[126] Baba N., Higashi Y., Kanekura T. (2013). Japanese black vinegar “Izumi” inhibits the proliferation of human squamous cell carcinoma cells via necroptosis. Nutr. Cancer.

[127] Seki T., Morimura S., Shigematsu T., Maeda H., Kida K. (2004). Antitumor activity of rice-shochu post-distillation slurry and vinegar produced from the post-distillation slurry via oral administration in a mouse model. Biofactors.

[128] Fukuyama N., Jujo S., Ito I., Shizuma T., Myojin K., Ishiwata K., Nagano M., Nakazawa H., Mori H. (2007). Kurozu moromimatsu inhibits tumor growth of Lovo cells in a mouse model in vivo. Nutrition.

[129] Shizuma T., Ishiwata K., Nagano M., Mori H., Fukuyama N. (2011). Protective effects of fermented rice vinegar sediment (*Kurozu moromimatsu*) in a diethylnitrosamine-induced hepatocellular carcinoma animal model. J. Clin. Biochem. Nutr..

[130] Xibin S., Meilan H., Moller H., Evans H.S., Dixin D., Wenjie D., Jianbang L. (2003). Risk factors for oesophageal cancer in Linzhou, China: A case-control study. Asian Pac. J. Cancer Prev..

[131] Huang L., Chen L., Gui Z.X., Liu S., Wei Z.J., Xu A.M. (2020). Preventable lifestyle and eating habits associated with gastric adenocarcinoma: A case-control study. J. Cancer.

[132] Radosavljević V., Janković S., Marinković J., Dokić M. (2004). Non-occupational risk factors for bladder cancer a case-control study. Tumori J..

[133] Yousefian M., Shakour N., Hosseinzadeh H., Hayes A.W., Hadizadeh F., Karimi G. (2019). The natural phenolic compounds as modulators of NADPH oxidases in hypertension. Phytomedicine.

[134] Na L., Chu X., Jiang S., Li C., Li G., He Y., Liu Y., Li Y., Sun C. (2016). Vinegar decreases blood pressure by down-regulating AT1R expression via the AMPK/PGC-1α/PPARγ pathway in spontaneously hypertensive rats. Eur. J. Nutr..

[135] Kondo S., Tayama K., Tsukamoto Y., Ikeda K., Yamori Y. (2001). Antihypertensive effects of acetic acid and vinegar on spontaneously hypertensive rats. Biosci. Biotechnol. Biochem..

[136] Nishikawa Y., Takata Y., Nagai Y., Mori T., Kawada T., Ishihara N. (2001). Antihypertensive effects of Kurosu extract, a traditional vinegar produced from unpolished rice, in the SHR rats. J. Jpn. Soc. Food Sci..

[137] Saito N., Segawa Y., Maruyama S., Yamaoka A., Hashimoto H., Osera T., Kurihara N. (2017). Abstract P196: Effect of combined oral intake of ginger extract and rice vinegar on blood pressure with 2-kidney, 1-clip renovascular hypertensive rats. Hypertension.

[138] Maruyama S., Segawa Y., Saito N., Yamaoka A., Hashimoto H., Osera T., Kurihara N. (2017). Abstract P198: Combination of *Undaria pinnatifida* sporophyll and vinegar remarkably decreases blood pressure in 2-kidney, 1-clip renovascular hypertensive rats. Hypertension.

[139] Tanaka H., Watanabe K., Ma M., Hirayama M., Kobayashi T., Oyama H., Sakaguchi Y., Kanda M., Kodama M., Aizawa Y. (2009). The effects of γ-aminobutyric acid, vinegar, and dried bonito on blood pressure in normotensive and mildly or moderately hypertensive volunteers. J. Clin. Biochem. Nutr..

[140] Kajimoto S., Ooshima Y., Tayama K. (2003). Effect of vinegar-added beverages on hypotensive action in healthy or mild hypertensive subjects (Japanese). J. Nutr. Food.

[141] Diana M., Quílez J., Rafecas M. (2014). Gamma-aminobutyric acid as a bioactive compound in foods: A review. J. Funct. Foods.

[142] Hayakawa K., Kimura M., Kamata K. (2002). Mechanism underlying γ-aminobutyric acid-induced antihypertensive effect in spontaneously hypertensive rats. Eur. J. Pharmacol..

[143] Matsubara F., Ueno H., Tadano K., Suyama T., Imaizumi K., Suzuki T., Magata K., Kikuchi N., Muneyuki K., Nakamichi N. (2002). Effects of GABA supplementation on blood pressure and safety in adults with mild hypertension. Jpn. Pharmacol. Ther..

[144] Chen C., Chen F. (2009). Study on the conditions to brew rice vinegar with high content of γ-amino butyric acid by response surface methodology. Food Bioprod. Process..

[145] Xia T., Yao J., Wang J., Zhang J., Wang M., Liu H., Song C., Ram A. (2018). Antioxidant activity and hepatoprotective activity of Shanxi aged vinegar in hydrogen peroxide-treated HepG-2 cells. Advances in Applied Biotechnology.

[146] Mayeux R., Stern Y. (2012). Epidemiology of Alzheimer disease. Cold Spring Harb. Perspect. Med..

[147] Tripathi S., Mazumder P.M. (2020). Apple cider vinegar (ACV) and their pharmacological approach towards Alzheimer’s disease (AD): A review. Indian J. Pharm. Educ. Res..

[148] Tripathi S., Kumari U., Mitra Mazumder P. (2020). Ameliorative effects of apple cider vinegar on neurological complications via regulation of oxidative stress markers. J. Food Biochem..

[149] Kanouchi H., Kakimoto T., Nakano H., Suzuki M., Nakai Y., Shiozaki K., Akikoka K., Otomaru K., Nagano M., Matsumoto M. (2016). The brewed rice vinegar Kurozu increases HSPA1A expression and ameliorates cognitive dysfunction in aged P8 mice. PLoS ONE.

[150] Cao L., Song X., Song Y., Bi J., Cong S., Yu C., Tan M. (2017). Fluorescent nanoparticles from mature vinegar: Their properties and interaction with dopamine. Food Funct..

[151] Loke C., Lee J., Sander S., Mei L., Farella M. (2016). Factors affecting intra-oral pH—A review. J. Oral Rehabil..

[152] Willershausen I., Weyer V., Schulte D., Lampe F., Buhre S., Willershausen B. (2014). In vitro study on dental erosion caused by different vinegar varieties using an electron microprobe. Clin. Lab..

[153] Zheng L.W., Li D.Z., Lu J.Z., Hu W., Chen D., Zhou X.D. (2014). Effects of vinegar on tooth bleaching and dental hard tissues in vitro. Yi Xue Ban.

[154] Anderson S., Gonzalez L.A., Jasbi P., Johnston C.S. (2020). Evidence that daily vinegar ingestion may contribute to erosive tooth wear in adults. J. Med. Food.

[155] Chung C.H. (2002). Corrosive oesophageal injury following vinegar ingestion. Hong Kong Med. J..

[156] Chang J., Han S.E., Paik S.S., Kim Y.J. (2020). Corrosive esophageal injury due to a commercial vinegar beverage in an adolescent. Clin. Endosc..

[157] Lhotta K., Hofle G., Gasser R., Finkenstedt G. (1998). Hypokalemia, hyperreninemia and osteoporosis in a patient ingesting large amounts of cider vinegar. Nephron.

[158] Varvarelis N., Khallafi H., Pappachen B., Krishnamurthy M. (2007). Natural therapies—When ignorance is not bliss!. J. Am. Geriatr. Soc..

[159] Irkoren S., Sivrioglu N. (2014). Unusual burn injury due to application of white vinegar and aspirin mixture. Int. Wound J..

[160] Feldstein S., Afshar M., Krakowski A.C. (2015). Chemical burn from vinegar following an internet-based protocol for self-removal of nevi. J. Clin. Aesthet. Dermatol..

[161] Benmeir P., Lusthaus S., Weinberg A., Neuman A., Eldad A. (1994). Facial chemical burn. Burns.

